# Altered crosstalk of bacterial lipopolysaccharide with immune cells in colorectal cancer compared to paired adjacent intestinal tissue

**DOI:** 10.1080/19490976.2026.2665878

**Published:** 2026-05-05

**Authors:** Åsa Walberg, Anna Maria Reuss, Reihane Ziadlou, Céline Mamie, Claudia Gottier, Anna White, Mohammadmilad Ameri, Marie-Charlotte Brüggen, Matthias Turina, Michaela Ramser, Paulina Wawrzyniak, Maria Walker, Luca Truscello, Adriano Aguzzi, Anne Müller, Barbara Hubeli, Yasser Morsy, Michael Scharl

**Affiliations:** a Department of Gastroenterology and Hepatology, University Hospital Zurich, University of Zurich, Zurich, Switzerland; b Institute of Neuropathology, University Hospital Zurich, University of Zurich, and ZNZ Neuroscience Center, Zurich, Switzerland; c Department of Dermatology, University of Zurich, Zurich, Switzerland; d Department of Visceral and Transplant Surgery, University Hospital Zurich, Zurich, Switzerland; e Institute for the Science of the Aging Brain (ISAB), St. Gallen, Switzerland; f Institute of Molecular Cancer Research, University of Zurich, Zurich, Switzerland; g Comprehensive Cancer Center Zurich, University Hospital Zurich, University of Zurich, Zurich, Switzerland

**Keywords:** Gut microbiota, colorectal cancer, bacterial-immune interactions, bacterial lipopolysaccharide (LPS), tumor microenvironment, spatial transcriptomics, imaging mass cytometry, 3D imaging, adapted iDISCO

## Abstract

Commensal bacteria play a crucial role in modulating human immune responses in the intestine. Under homeostatic conditions, the gut microbiota is tightly regulated by interactions with the mucosal immune system. However, colorectal cancer (CRC) is characterized by an imbalance in bacterial composition and bacterial translocation across the intestinal barrier. The spatial distribution of bacteria and their interactions with immune cells in CRC tumors are poorly understood. By applying 3D light-sheet imaging, spatial transcriptomics, and imaging mass cytometry to patient-derived CRC and adjacent intestinal tissue, bacterial lipopolysaccharide (LPS) can be visualized alongside immune cells and vessels. The results showed regional bacterial LPS accumulation and colocalization with distinct immune cell subsets. In CRC-adjacent tissue, bacterial LPS is mainly associated with CD11c^+^ dendritic cells, CD15^+^ neutrophils, and CD163^+^ macrophages. In matched CRC tissue, the number and LPS colocalization of CD163^+^ macrophages and CD11c^+^ dendritic cells decreased, while CD15^+^ neutrophils and their colocalization with LPS increased. Notably, immune cell composition and immune cell‒bacteria interactions differ between tumors and adjacent tissue, offering insights into host‒microbiota dynamics and mechanistic interactions.

## Introduction

The human gut microbiome, as well as intra-tumoral bacteria, are pivotal in the development and progression of cancer.^1-7^ Microbiota‒immune cell interactions in the intestine require both tolerance for commensal bacteria and defense mechanisms against harmful pathogens. Under homeostatic conditions, several defense mechanisms control and compartmentalize the gut bacteria to the mucus layer that covers the intestinal epithelium.^8^ In addition to epithelial and mucosal barriers, both innate and adaptive immune mechanisms contribute to the regulation of the microbial composition. For instance, dendritic cells (DCs) continuously sample and present bacterial antigens to stimulate antigen-specific T cell responses. Goblet cells in the epithelium can transcytose gut luminal contents, including bacterial antigens, resulting in T cell-mediated antigen-specific tolerance.^9^ In contrast, B-/plasma cells contribute to intestinal homeostasis through the production of secretory IgA.^10^

However, in cancer patients, the gut microbiome is frequently characterized by alterations in bacterial composition and function.^11^ These alterations impact the gut barrier and immune cell responses.^11-13^ Furthermore, certain bacterial species were found in particularly large numbers within the colorectal cancer (CRC) tissue.^3^^,^^14^^,^^15^ Though it remains unclear how bacteria infiltrate cancerous tissues, some reports suggest that the CRC-associated bacterium, *Fusobacterium nucleatum*, can translocate via a hematological route to the CRC tumors.^16^^,^^17^ Particularly in CRC, an apparent origin of bacteria detectable within the tumor microenvironment (TME) is the gut lumen, from where bacterial translocation likely originates.^18^ Alterations in the TME and disruption of the epithelial and mucosal barrier, as typically found in CRC, may favor the enrichment of selected bacteria at tumor sites.^19^ Of note, both pro-tumorigenic and anti-tumorigenic effects of bacteria have been shown in animal models and human studies.^5^^,^^20^^,^^21^ However, the specific bacteria-immune interactions in tumor and tumor-adjacent tissue, as well as the spatial location of bacteria in tumors, are not well characterized. Bacteria have been described mainly intracellularly in cancer cells and immune cells within tumor tissue, but also extracellularly in cancer tissue of various tumor types.^22-25^ In CRC, bacteria have been detected within epithelial cells at the primary tumor site and the metastatic liver lesion.^3^^,^^25^ Furthermore, biofilm formations, i.e., large aggregates of bacteria, have been found in the unaffected adjacent colon as well as in the CRC tissue.^3^^,^^26-28^ In our present study, we analyzed paired CRC tissue and adjacent intestinal tissue from the same patient. We focused on bacterial lipopolysaccharide (LPS) as a general marker for gram-negative bacteria, such as *Fusobacterium nucleatum* or *Bacteroides fragilis*, to assess localized alterations in microbiota‒immune interactions in patients with CRC. A better characterization and particularly understanding of bacterial–immune cell interactions in patient-derived tissue will not only help to improve our understanding of the regions populated by bacteria in CRC tumors, but also provide important insights into the mechanistic effects of bacteria on CRC pathogenesis, which in turn could contribute to our understanding of bacteria-based cancer therapies.

## Results

### 3D imaging demonstrates aggregates of bacterial LPS and colocalization with immune cells in CRC tissue

**Figure 1.**
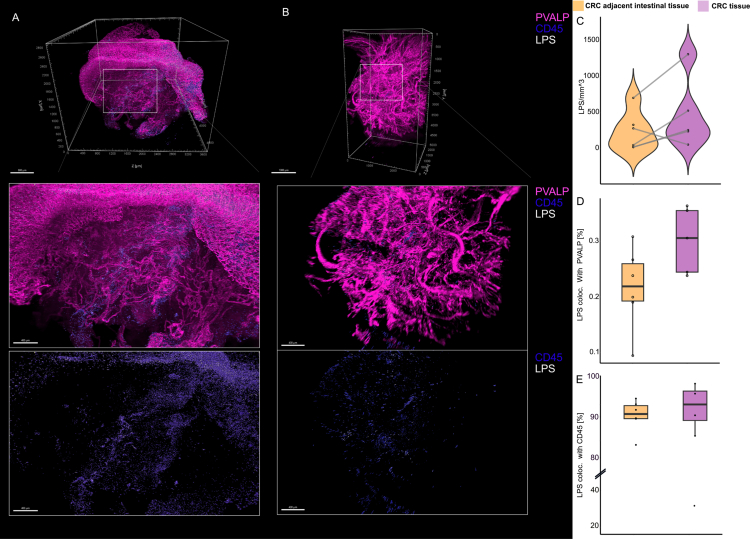
Global overview of bacteria-immune cell interactions in CRC-adjacent intestinal tissue and CRC tissue using 3D histology. (A) Volume rendering of the vasculature (PVALP), immune cells (CD45), and bacterial lipopolysaccharide (LPS) in CRC-adjacent intestinal tissue and with zoom-ins (white boxes)(B) Volume rendering of CRC tissue stained with the same markers as described in (A), with zoom-ins (white boxes) . (C) Violin plot presenting the ratio of detected bacterial LPS per mm³ in CRC-adjacent intestinal tissue (orange) and CRC tissue (violet). Lines connect the CRC-adjacent intestinal tissue and the CRC tissue from the same patient. (D, E) Percentage of bacterial LPS colocalized (coloc.) with the vasculature (D) and immune cells (E) in CRC-adjacent intestinal tissue(orange) and CRC tissue (violet). Boxes represent the interquartile range, with the median indicated by a horizontal line. *n* = 6 CRC-adjacent intestinal tissue samples and *n* = 5 matched tumor samples.

To obtain a global overview of bacterial and immune cell localization within the CRC-adjacent intestinal tissue and the CRC tissue, we applied organic solvent-based tissue clearing and immunolabeling to approximately 5 mm³-sized patient-derived tissue samples. We applied bacterial LPS as a marker for gram-negative bacteria, CD45 as a general immune cell marker, and PVALP as a marker for the vasculature. In total, six patients with CRC were included in the analysis, comprising six CRC-adjacent intestinal tissue samples and five matched CRC samples from the same patients (patient details are described in Table S1). In the CRC-adjacent intestinal tissue, bacterial LPS was detected mainly in the epithelial cell layer, likely deriving from bacteria-rich mucus remnants (Figure 1A, S1, S2A, B, Suppl. Video 1). Additionally, in 3/6 CRC-adjacent intestinal tissue samples, we observed bacterial LPS translocation into subepithelial/submucosal layers (Figure S1). In contrast, in CRC tissues, we observed selective regions of LPS, indicative of bacterial abundance, rather than an even distribution across the whole tissue (Figure 1B, S3A, B, Suppl. Video 2). The comparison between the matched CRC tissues and the CRC-adjacent intestinal tissues revealed an increase in bacterial LPS concentration per volume (mm^3^) in 4/5 CRC tissues (Figure 1C). However, the extent of increase varied substantially between patients, indicating high inter-patient variability in LPS accumulation. Notably, only a few of the bacterial LPS signals colocalized with blood vessels (Figure 1D). The mean colocalization of bacterial LPS with PVALP, indicative of blood vessels, was 0.3 ± 0.05% SD in CRC tissue and slightly lower (0.2 ± 0.07% SD) in CRC-adjacent intestinal tissue. Additionally, our results indicated a higher number of blood vessels per mm³ in the tumor tissue (5817 ± 2839 SD) than in the CRC-adjacent intestinal tissues (2602 ± 1163 SD) (Figure S4A, B). Across all samples, we obtained high percentages of colocalization of bacterial LPS with CD45+ immune cells (Figure 1E, Figure S4C, D). The mean of LPS–CD45 colocalization was 90% in CRC-adjacent intestinal tissue samples and 80% in CRC tissue samples. Overall, our 3D staining revealed an increase in bacterial LPS within the CRC tissue, co-localizing with immune cells.

### Spatial transcriptomics analysis detects inflamed epithelial cells as increased in paired CRC tissue compared to adjacent intestinal tissue

**Figure 2.**
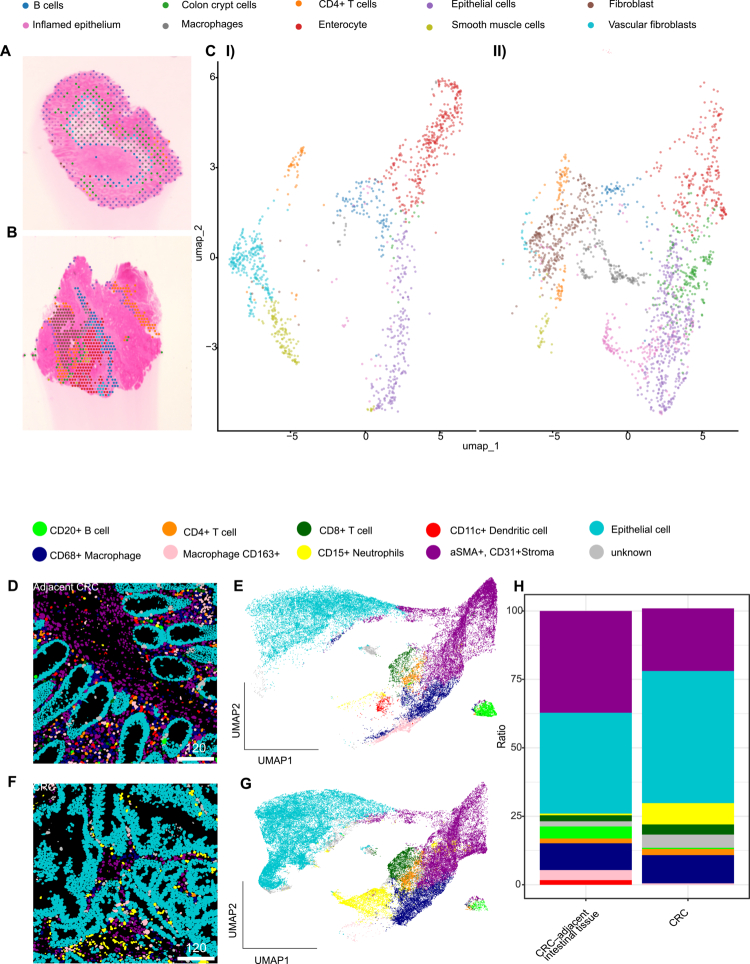
Immune cell types were defined in 5 µm CRC-adjacent intestinal tissue and CRC tissue. (A) Representative H&E staining and spatial annotation of 10 cell types detected in 3 CRC-adjacent intestinal tissue and (B) matched CRC sections using spatial transcriptomics. (C) UMAP plot showing the distributions of 10 distinct cell types from (I) all CRC-adjacent intestinal tissue and (II) CRC samples detected in spatial transcriptomics. (D): Representative imaging mass cytometry (IMC) image of 10 different cell types in CRC-adjacent intestinal tissue, and (E): respective UMAP showing cell type clustering. (F): Representative IMC image of cell types in CRC, and (G): Respective UMAP representing clustering of cell types. (H): Stacked bar plots represent the mean of cell type proportions across all patients (*n* = 9 CRC and CRC-adjacent intestinal tissue). Data for each biological replicate represents the average of two technical replicates (images) per patient. Statistical significance was determined via a paired Wilcoxon signed-rank test on patient-averaged proportions, with *p*-values adjusted for multiple comparisons using the Benjamini‒Hochberg method.

To gain an overview of the immune cell landscape in CRC tissue and paired adjacent intestinal tissue from the same patients, we next applied spatial transcriptomics to tissues from three patients with CRC (Figure S5A shows histopathological staining (H&E) of these patients). We detected 10 different cluster cell types within the spatial transcriptomics data of both CRC-adjacent intestinal tissue and CRC tissue (Figure 2A, B, C, Figure S5B) for the genes expressed within these cell clusters. Particularly, an increased number of cells related to the cluster of inflamed epithelial cells was detected in CRC tissue compared to CRC-adjacent intestinal tissue. This cluster of cells expressed a few epithelial cell markers, but additionally several neutrophil-associated markers, such as S100A8 and S100A9, IL1B and MMP9, together with the neutrophil-recruiting chemokine CXCL8. Such clusters have been described previously in intestinal tissue.^29^ In addition, CRC tissue samples exhibited a higher abundance of macrophages compared to their matched CRC-adjacent intestinal tissues.

### Imaging mass cytometry detects a decrease in antigen-presenting cells, accompanied by an increase in neutrophils in CRC tissue compared to adjacent tissue

To understand which immune cells are interacting with bacterial LPS in CRC-adjacent intestinal tissue and in CRC tissue, we applied imaging mass cytometry (IMC) using a panel with 31 different immune cell markers as well as bacterial LPS (Suppl. Table S2). Two regions (each 600 µm × 600 µm) per CRC-adjacent intestinal tissue (Figure 2D, E) as well as CRC tissue (Figure 2F, G) from nine patients, including the patients with CRC studied above, were analyzed (patient details can be found in Suppl. Table S1 and H&E staining of tissue samples are displayed in Supplementary Figure S6). The mean cell count per image was lower in the CRC-adjacent intestinal tissue samples (2447 ± 321 SD) than in tumor samples (3140 ± 703 SD) (Figure S7A). Overall, 44,053 cells were identified in all adjacent tissues and 56522 cells in all CRC tissues (Figure S7B). To define the cell types in CRC-adjacent intestinal tissues and CRC tissues, we applied an unsupervised clustering approach using FlowSOM, determining cell types based on similar marker expression levels found within a cluster of cells (Suppl. Table S3, Figures S8, S9). The frequencies of cell types from each of the two regions scanned per patient and tissue are visualized for CRC-adjacent tissue and CRC tissue in Supplementary Figure S10A and B, respectively (Figure S10A). The most abundant cell populations were epithelial cells in both CRC-adjacent intestinal tissues and CRC tissues, with average 36.8% ± 1.12 SEM and 48.2% ± 8.1 SEM, respectively (Figure 2H, Figure S10A). There were comparable frequencies of CD68+  macrophages (9.7 ± 0.9% SEM and 10.3 ± 1.69% SEM), CD4+  T cells (1.7 ± 0.4% SEM and 2.2 ± 0.3% SEM), and CD8+  T cells (2.1 ± 0.5% and 3.7 ± 1.02% SEM) in both CRC-adjacent intestinal tissues and CRC tissues. Additionally, we detected a distinct CD68+ macrophage population, which was positive for CD163. CD163 is a plasma membrane glycoprotein and member of the scavenger receptor cysteine-rich (SRCR) superfamily class B, which is highly expressed in tissue-resident macrophages.^30^ Here, the CD163+ macrophage population was significantly more abundant in CRC-adjacent intestinal tissue (3.6 ± 0.8%SEM) compared to CRC tissue (0.5v%0.1 SEM). CD20+ B cells were decreased in CRC tissue (0.4% ± 0.1 SEM) compared to CRC-adjacent intestinal tissue (4.3% ± 2.1 SEM), and similarly, αSMA+  and CD31+ stroma cells were found at a significantly lower ratio in CRC tissue (22.8% ± 4.1) than in CRC-adjacent intestinal tissue (37.2% ± 2.2 SEM). CD11c+ dendritic cells (1.6% ± 0.4 SEM) were the least abundant cell type in CRC-adjacent intestinal tissue. Notably, there were too few CD11c+ dendritic cells in the CRC tissue to form such a cluster cell type, resulting in the absence of this cell type in the CRC tissue based on these specific markers used in IMC. In contrast, CD15+  neutrophils were significantly increased in CRC (7.7% ± 1.8 SEM) compared to CRC adjacent intestinal tissue (0.6% ± 0.1 SEM). Interestingly, these neutrophils additionally expressed Granzyme B. Confirming our findings, our spatial transcriptomics and IMC data revealed overlapping cell populations, with B cells, epithelial cells, and inflamed epithelium showing consistent trends across both approaches, even though additional cell types were uniquely detected by each method (Figures S11A and S11B).

### Distinct bacterial LPS-immune cell crosstalk in CRC-adjacent intestinal tissue compared to CRC tissue

Having identified the cell types in the CRC-adjacent intestinal and CRC tissues, we next investigated potential interactions between distinct human host cell types and bacterial LPS. We defined a cell as associated with bacterial LPS (LPS+) if the expression of the bacterial LPS marker within a cell exceeded a 90th percentile cutoff. Therefore, we chose a strict cutoff to minimize any potential background or overspill from different channels. Among the cells rated as associated with bacterial LPS, we observed a higher relative expression of activation markers, including CD25 in B cells, CD69 in CD4+  and CD8+ T cells, as well as CD163 in macrophages (Figure S12).

**Figure 3.**
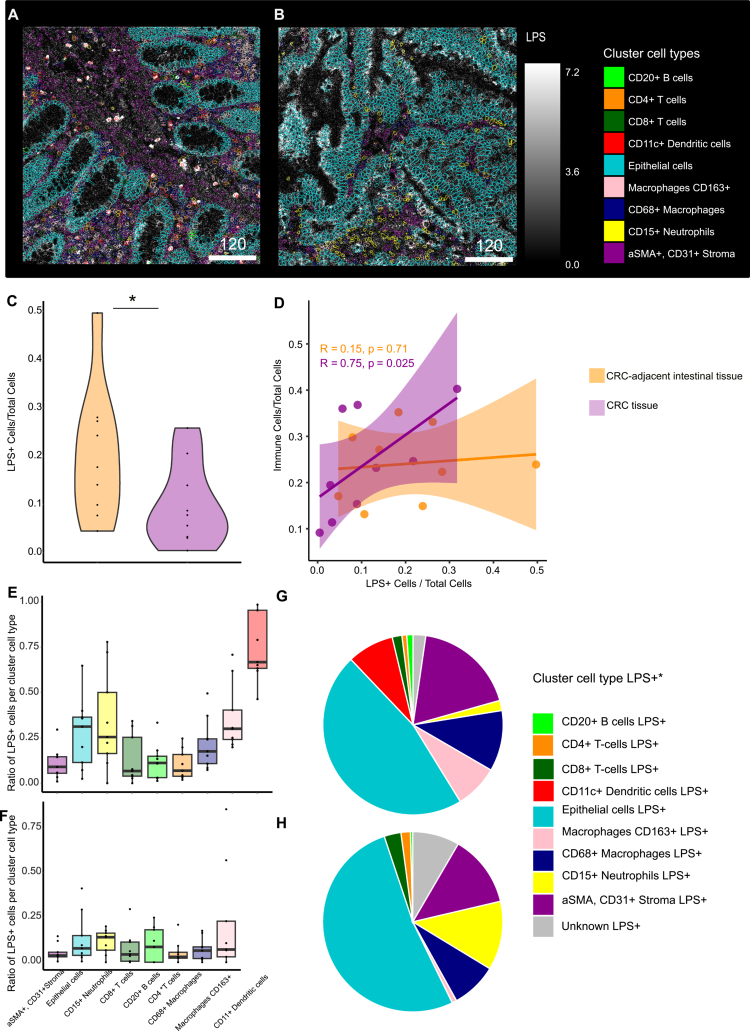
Differences in LPS-associated cell types between paired CRC adjacent intestinal tissues and CRC using IMC. (A, B) Representative images of LPS expression and associated cell types in CRC-adjacent intestinal tissue (A) and CRC (B). (C) Violin plots showing the distribution of LPS⁺ cells in CRC-adjacent intestinal tissue (orange) and matched CRC (purple) from 9 patients. (D) Spearman rank correlation showing the relationship between immune cell infiltration and the LPS+ cell ratio. (E) Ratio of LPS⁺ cells per cell type cluster in CRC adjacent intestinal tissue and (F) CRC tissue. The Kruskal‒Wallis test followed by Dunn's post-hoc test with Benjamini‒Hochberg correction was used to determine potential differences in the ratios of LPS+  cells among different cell types in CRC adjacent intestinal tissue and CRC. (G) A pie chart showing the ratio of each cell type within the LPS⁺ cell population in adjacent intestinal tissue, and (H) CRC tissue. Differences in the ratio of LPS+  cell types between CRC-adjacent intestinal tissue and CRC tissue were tested using the Wilcoxon signed-rank test for each cell type, with Bonferroni correction for multiple comparisons. Significant differences were considered at *p* < 0.05. ns = not significant (*p* > 0.05). Each data point represents the average of the two images analyzed per patient sample for paired CRC adjacent intestinal tissue and CRC tissue (*n* = 9), respectively.

By IMC, we observed that LPS signals appeared as more isolated dot-like spots in the CRC-adjacent intestinal tissues, in contrast to a more diffuse signal in CRC tissues (Figure 3A, B).

To assess the distribution of LPS across CRC and CRC-adjacent intestinal tissue, we compared both the absolute abundance and the relative frequency of LPS+  cells in patient-matched samples (*n* = 9). We observed a significant difference (*p*-value = 0.003) between the relative number of LPS+  cells to all cells per patient in the CRC-adjacent intestinal tissue and the CRC tissue (Figure 3C). Interestingly, the absolute count of LPS+  cells per patient remained comparable between CRC and CRC-adjacent intestinal tissue (*p*-value = 0.16) (Figure S13). Subsequently, we investigated the relationship between the abundance of bacterial LPS+  cells and the ratio of immune cells (Figures 3D, S14B). Spearman's rank correlation of tumor samples revealed a positive relationship between the ratio of LPS+  cells and immune cells to all cells (rho = 0.75, *p*-value = 0.02), indicating a trend towards increased immune cell infiltration with a higher overall count of LPS+ immune cells in the CRC tissue. In contrast, this association was not observed in the CRC-adjacent intestinal tissue (rho = 0.15, *p*-value = 0.70), indicating a tumor-specific interaction. Interestingly, for the number of epithelial cells, we found an inverse relationship with the number of LPS+  cells (Figure S14A, C, D).

These findings suggest that higher proportions of LPS+  cells in CRC tumors may coincide with an increased immune cell infiltration into the CRC tissue. Finally, we assessed how frequently individual cell types were associated with bacterial LPS. Within the CRC-adjacent intestinal tissue, the majority of the dendritic cells were associated with bacterial LPS (74% ± 0.06 SEM) (Figure 3E). Similarly, on average, 34% ± 0.08 SEM of all neutrophils and 35% ± 0.06 SEM of all CD163+  macrophages in CRC-adjacent intestinal tissue were LPS+. For these three cell types, the Kruskal–Wallis test showed significant differences compared to other cell types. For the CRC tissue, the highest ratio of LPS+  cells per cell type was 21% ± 0.1 SEM in CD163+  macrophages, 11% ± 0.02 SEM in CD15+  neutrophils, and 13% ± 0.02 SEM in epithelial cells (Figure 3F). Kruskal‒Wallis analysis did not show significant differences between any of the cell types within the CRC tissue.

Additionally, we investigated the cell type composition among all LPS+  cells in the CRC-adjacent intestinal tissue versus the CRC tissue to determine which immune cell types were the most represented among them. Among all LPS+  cells per image, the fraction of dendritic cells and CD163+  macrophages was higher in the CRC-adjacent intestinal tissue (Figure 3G) compared to the CRC tissue (Figure 3H). Additionally, a higher ratio of epithelial cells and neutrophils was detected among LPS+  cells per image in CRC compared to CRC-adjacent intestinal tissue. CD163+ macrophages, and CD11c+  dendritic cells differed significantly between LPS+  cells per image from CRC compared to the CRC-adjacent intestinal tissue.

To support our findings, we filtered and re-examined metagenomic data from the nine CRC patients analyzed in this study from our larger previously published dataset.^18^ CRC-adjacent intestinal tissue and CRC tissue samples from the same patients showed no significant differences in the overall composition of the bacterial genera (Figure S15A). However, we detected an increased abundance of the CRC-associated *Fusobacterium nucleatum* in the CRC tissue, which is in line with several previous findings, and this trend did not reach statistical significance in our study (Figure S15B). Notably, *Lachnoclostridium* sp. YL32 was the only species that showed a statistically significant difference in relative abundance between CRC and CRC-adjacent intestinal tissues (Figure S15C). This finding is consistent with previous reports indicating an enrichment of *Lachnoclostridium* sp. YL32 in patients with colorectal adenomas compared to healthy controls.^31^ Despite this taxonomic shift, the relative abundance of *Lachnoclostridium sp. YL32* did not correlate significantly with the LPS levels detected by IMC (Figure S15D). Furthermore, we did not find a significant correlation between the total number of gram-positive or gram-negative species identified in the metagenomic dataset and the quantity of LPS detected by the IMC (Figure S16). Interestingly, however, selective species did show a correlation with IMC-detected LPS levels, including *Anaerostipes caccae, Collinsella aerofaciens, and Lachrimispora saccharoltytica,* along with six additional species in CRC-adjacent intestinal tissue, as well as *Subdoligranulum variabile* and *Lachrimispora saccharoltytica* in the CRC tissue (Figure S17).

### A distinct spatial context of LPS+ -associated immune cells differentiates CRC-adjacent intestinal tissue from paired CRC tissue

**Figure 4.**
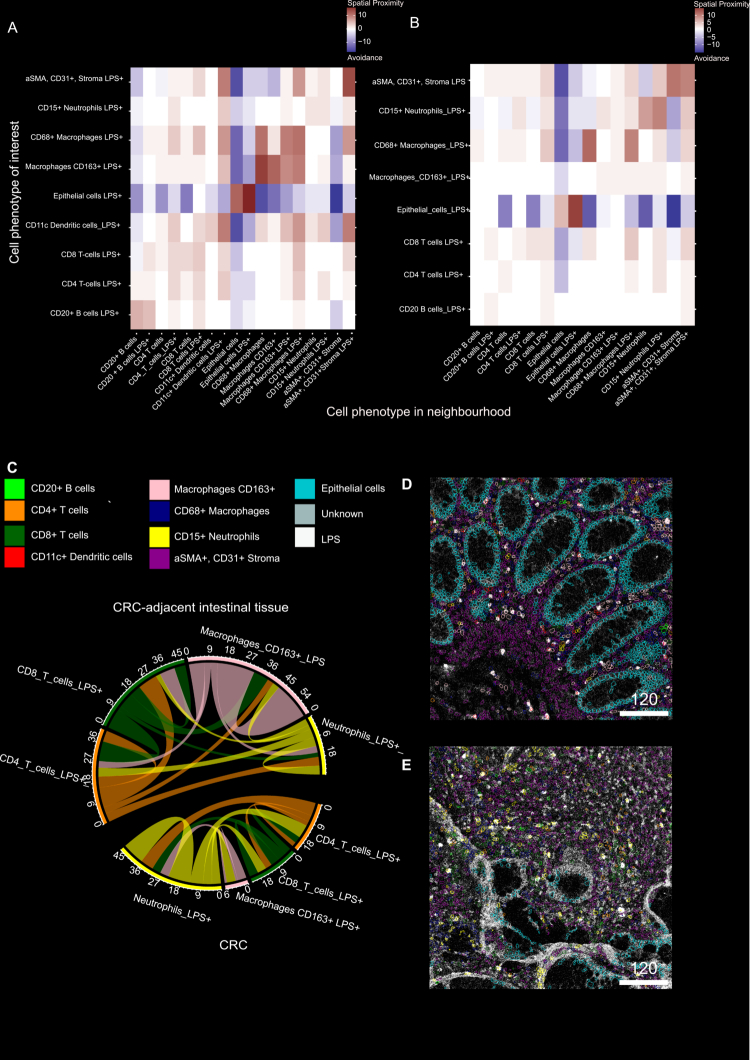
Differences in the spatial context of LPS-associated cells in CRC adjacent intestinal tissue and CRC tissue via IMC. (A, B) Heatmap of spatial proximity observed in 9 (A) CRC-adjacent intestinal tissue (B) and 9 CRC tissues, 2 images were analyzed per individual patient tissue. Each cell type in each row is significantly neighbored (red) or avoided (blue) by the cell type in each column. Significance was determined using permutation test (*P* < 0.01). (C) Chord diagram summarizing the frequency of observed spatial proximity of CD15+  neutrophils, CD163+  macrophages and CD4+  and CD8+  T-cells in CRC-adjacent intestinal tissue and CRC tissue. (D) Exemplary IMC images showing CD15 neutrophil, CD163+ macrophages, CD8 and CD4 T cells spatial proximities in CRC-adjacent intestinal tissue, and (E) CRC tissue.

We observed differences in the ratios of specific cell types in CRC tissue compared to CRC-adjacent intestinal tissue and, consequently, changes in bacterial LPS interactions with immune cells within CRC tissue. Next, we investigated the surrounding spatial context of cells associated with bacterial LPS in CRC-adjacent intestinal and CRC tissues. All cell types showed self-interactions, suggesting spatial clustering. Epithelial cells were compartmentalized in CRC-adjacent intestinal tissue and CRC tissue and were not found in spatial proximity to other cell types. In CRC-adjacent intestinal tissue, LPS+ CD15+  neutrophils were found in spatial proximity to LPS+ CD4+  T cells, LPS+ CD11c+  dendritic cells, and LPS+ CD163+  macrophages (Figure 4A). In CRC tissue, LPS+ CD15+  neutrophils were additionally located in close proximity to LPS+ CD8+  T cells and LPS+  and LPS- CD68+  macrophages and CD163+  macrophages. Additionally, LPS+  neutrophils suggest stronger spatial clustering in CRC tissue than in CRC-adjacent intestinal tissue.

LPS+ CD163+  macrophages were found in spatial proximity to LPS+ and LPS-CD4+ T cells and CD8+ T cells, as well as to LPS+ CD15+ neutrophils and LPS+ and LPS-CD68+ macrophages in CRC-adjacent intestinal tissue. In contrast, there was a lack of spatial proximity between LPS+ CD163+  macrophages and LPS+  and LPS-CD4+  and CD8+  T cells in CRC tissue (Figure 4B), but increased spatial proximity of LPS+ CD163+  to CD15+  neutrophils. However, the cellular proximity between LPS+ CD15+  neutrophils and LPS+ CD8+  and CD4+ T cells was less often found in CRC-adjacent intestinal tissue (Figure 4C, D) compared to CRC tissue (Figure 4C, E).

Overall, the main distinction of LPS+  cell proximity found between CRC-adjacent intestinal tissue and CRC tissue was the loss of spatial proximity of LPS+ CD163 macrophages and dendritic cells to T cells, while, in contrast, more frequent spatial proximity was found of LPS+ neutrophils to T cells in the CRC tissue.

Overall, we observed alterations in the composition of LPS–immune cells and in the spatial context, whereas no significant differences in bacterial profiles were detected between paired CRC and CRC-adjacent intestinal tissues.

## Discussion

Our study provides evidence for shifts in the location of bacteria-derived LPS and bacteria‒immune cell interactions from the CRC-adjacent intestinal tissue to the paired CRC TME. Using 3D histology of cleared ~5 mm^3^ tissues, we showed that bacteria-derived LPS can be found translocated to the subepithelial/submucosal tissue, both in the tumor as well as in the CRC adjacent intestinal tissue. Regional abundance of bacterial LPS was characteristic of CRC tissue. Additionally, we observed an overall higher bacterial biomass per mm³ in the CRC tissue compared to the CRC-adjacent intestinal tissue. This finding is in line with recent data in which higher bacterial biomass was detected in CRC tissue compared to paired normal and healthy tissues.^27^^,^^32^ However, we did not detect a significantly higher amount of bacterial LPS in the CRC tissue in the IMC dataset, highlighting the heterogeneous distribution of bacteria-derived LPS in tumors. The localized patches of bacteria, as seen in our 3D imaging data, emphasize the importance of whole biopsy/tissue resection analysis for microbiome studies. Thus, standard 2D histology of 5 µm-thick FFPE sections might miss bacterial presence in tumor samples unless a multitude of serial sections are analyzed.

In the 3D histological data, we observed a high level of colocalization of bacterial LPS with immune cells. In line with these findings, we found a significant relationship between the number of immune cells infiltrating into tumor tissue and the number of LPS+  cells in CRC tissue using IMC. Interestingly, the number of epithelial cells showed an inverse relationship to the number of LPS+  cells, which is consistent with recent findings showing that bacteria-rich regions in CRC are characterized by reduced cancer epithelial cell density and proliferation.^33^

The investigation of immune cell types via spatial transcriptomics and IMC showed alterations in specific immune cell numbers in CRC tissue. First, CD11c⁺ dendritic cells are frequently associated with LPS in CRC-adjacent intestinal tissue; however, this specific dendritic cell subtype was not detected within tumor samples, which might be due to the impact of tumor-secreting factors on the development and numbers of this dendritic cell subtype within tumors.^18^ Furthermore, we observed a lower amount of CD163+  macrophages in tumor tissue compared to matched adjacent colon tissue, which is in line with findings in the literature.^22^ Interestingly, CD163+ expressing macrophages were found in higher numbers in inflamed tissue; for example, over-expression of CD163 was found in areas of active inflammation in IBD, and the expression of both CD163 RNA and protein was increased in IBD in comparison to normal controls.^23^ Here, we found that in the CRC-adjacent intestinal tissue samples, CD163+  macrophages were frequently associated with bacterial LPS. In line with this LPS+  association, CD163 can function as a macrophage receptor for bacteria, acting as an inducer of local inflammation during bacterial infection.^24^

Next, we observed that CD15+  neutrophils are already present at the CRC-adjacent intestinal barrier site, a cell type not prevalently found in colon tissue under homeostatic conditions.^34^ In fact, crosstalk with the commensal gut microbiota has been found to suppress neutrophil recruitment during intestinal health.^35^ The presence of neutrophils in the CRC-adjacent intestinal tissue could reflect an impaired gut barrier integrity.^36^ A weakened gut barrier might subsequently enable bacterial translocation to the underlying layers, as supported by our 3D histology data of CRC-adjacent intestinal tissue. In line with this, the PVALP protein visualized in our 3D imaging dataset is also a marker of an impaired gut vascular barrier and was previously reported to be increasingly found in CRC and CRC-adjacent intestinal tissue, and not detected in healthy colon.^12^ Additionally, as seen in our study and supported by findings in the literature, neutrophils have been increasingly found within the CRC tissue.^32^ Interestingly, the neutrophil population identified in our IMC data set co-expresses granzyme B, which is consistent with a previous study where this cell type was detected in human colon tumors and rodent models.^37^ Lipid A analog, the lipid part of LPS, induced the release of granzyme B by these neutrophils, leading to tumor cell apoptosis.^37^ Hence, the CD15+ granzyme B-expressing cell subtype population might represent an anti-tumor population of neutrophils, suggesting a pro-inflammatory N1-like type.^37^ While we did not measure functional anti-tumor activity of neutrophils in this study, our spatial transcriptomics data also revealed that pro-inflammatory, activated neutrophil-associated genes were upregulated explicitly in CRC tissue, further supporting an N1-like polarization of detected neutrophils in the CRC microenvironment.

In contrast, tumor-associated neutrophils might also induce the shielding of cytotoxic immune cells from tumors due to their neutrophil extracellular trap (NET) formation.^20^^,^^38^ In a recent study, immature CD66b+ cells, which are also used as a neutrophil marker, were found in immunosuppressive regions of CRC.^15^ Here, we see a higher association of CD15+ neutrophils with CD4+ T cells and CD8+ T cells in tumor tissue compared to the adjacent colon. Neutrophil interactions with T cells can lead to both suppression and apoptosis as well as activation.^21^^,^^39-42^

Interestingly, the depletion of CD11c⁺ dendritic cells and CD163⁺ macrophages in CRC tissue, together with an increased neutrophil presence, suggests a shift toward an alternative mechanism of antigen presentation to T cells. Neutrophils have been shown to activate autologous antigen-specific memory T cells *in vitro.* Furthermore, these neutrophil subsets have been shown to induce potent anti-tumor immunity in murine models.^43^ Thus, in addition to spatial proximity, a more detailed analysis of the neutrophils and T cell markers could provide insights into their functional role and whether immunosuppressive or pro-inflammatory effects occur after interactions of activated neutrophils and T cells in CRC.

Consistent with our findings, an increased infiltration of neutrophils and CD4⁺ T cells in CRC tissue compared to healthy mucosa was moderately associated with the presence of specific bacterial strains, such as *Bacteroides fragilis* and *Fusobacterium nucleatum*, and was more strongly linked to a general increase in overall bacterial activity beyond these core pathogens.^27^ Metagenomic analysis of paired CRC and adjacent colon tissue from the same patients revealed that only one gram-positive bacterial species was differentially abundant. Thus, rather than individual bacterial species driving immune cell recruitment, the overall presence of translocated bacteria, bacteria-derived LPS, or other bacterial products may shape the immune landscape within the CRC tissue.

Overall, our findings contribute to the understanding of the heterogeneous TME and the bacteria as a component of it. We observed an inflammatory phenotype of the CRC tissue, accompanied by heterogeneous bacterial abundance and immune cell interactions in the CRC tissue compared to paired adjacent non-tumor tissue. Moreover, distinct changes in specific immune cell populations and their associations with bacterial LPS were identified in CRC tumors. A deeper understanding of these bacteria–immune cell interactions will be essential for understanding CRC pathogenesis and ultimately advancing bacteria-based therapeutic strategies in CRC.

## Supplementary Material

Supplementary Material Walberg_et_al_Suppl_Material_Altered_Crosstalk_clean.docx

Supplementary Material Gut Microbes Suppl videos.zip

Supplementary Material Figures_Supplement_reduced.pdf

## Resource availability

Raw TIFF image files generated from 3D histology and IMC experiments have been deposited at figshare https://doi.org/10.6084/m9.figshare.31054639 and are publicly available as of the date of publication. Metagenomic sequencing data from 9 CRC patients constitute a subset of a broader microbiome analysis previously published^18^ https://www.ncbi.nlm.nih.gov/bioproject/PRJNA1024674. Their respective Patient ID can be found in the Supplementary Table S4. This study did not generate new unique reagents or report original code. Requests for further information and resources should be directed to and will be fulfilled by the lead contact (Michael.scharl@usz.ch).

## References

[1] Hanahan D. Hallmarks of cancer: new dimensions. Cancer Discovery. 2022;12:31–46. doi: 10.1158/2159-8290.CD-21-1059. 35022204

[2] Kostic AD, Chun E, Robertson L, Glickman JN, Gallini CA, Michaud M, Clancy TE, Chung DC, Lochhead P, Hold GL, et al. Fusobacterium nucleatum potentiates intestinal tumorigenesis and modulates the tumor-immune microenvironment. Cell Host Microbe. 2013;14:207–215. doi: 10.1016/j.chom.2013.07.007. 23954159 PMC3772512

[3] Galeano Niño JL, Wu H, LaCourse KD, Kempchinsky AG, Baryiames A, Barber B, Futran N, Houlton J, Sather C, Sicinska E, et al. Effect of the intratumoral microbiota on spatial and cellular heterogeneity in cancer. Nature. 2022;611:810–817. doi: 10.1038/s41586-022-05435-0. 36385528 PMC9684076

[4] Pleguezuelos-Manzano C, Puschhof J, Rosendahl Huber A, Van Hoeck A, Wood HM, Nomburg J, Gurjao C, Manders F, Dalmasso G, Stege PB, et al. Mutational signature in colorectal cancer caused by genotoxic pks+ E. Coli. Nature. 2020;580:269–273. doi: 10.1038/s41586-020-2080-8. 32106218 PMC8142898

[5] Wu S, Rhee K-J, Albesiano E, Rabizadeh S, Wu X, Yen H-R, Huso DL, Brancati FL, Wick E, McAllister F, et al. A human colonic commensal promotes colon tumorigenesis via activation of T helper type 17 T cell responses. Nat Med. 2009;15:1016–1022. doi: 10.1038/nm.2015. 19701202 PMC3034219

[6] Wong SH, Zhao L, Zhang X, Nakatsu G, Han J, Xu W, Xiao X, Kwong TNY, Tsoi H, Wu WKK, et al. Gavage of fecal samples from patients with colorectal cancer promotes intestinal carcinogenesis in germ-free and conventional mice. Gastroenterology. 2017;153:1621–1633 e1626. doi: 10.1053/j.gastro.2017.08.022. 28823860

[7] Gopalakrishnan V, Spencer CN, Nezi L, Reuben A, Andrews MC, Karpinets TV, Prieto PA, Vicente D, Hoffman K, Wei SC, et al. Gut microbiome modulates response to anti–PD-1 immunotherapy in melanoma patients. Sci. 2018;359:97–103. doi: 10.1126/science.aan4236. PMC5827966 29097493

[8] Johansson MEV, Larsson JMH, Hansson GC. The two mucus layers of colon are organized by the MUC2 mucin, whereas the outer layer is a legislator of host–microbial interactions. Proc Natl Acad Sci USA. 2011;108:4659–4665. doi: 10.1073/pnas.1006451107. 20615996 PMC3063600

[9] Gustafsson JK, Davis JE, Rappai T, McDonald KG, Kulkarni DH, Knoop KA, Hogan SP, Fitzpatrick JA, Lencer WI, Newberry RD. Intestinal goblet cells sample and deliver lumenal antigens by regulated endocytic uptake and transcytosis. eLife. 2021;10:e67292. doi: 10.7554/eLife.67292. 34677124 PMC8594945

[10] Liu Y, Rhoads J. Communication between B-Cells and microbiota for the maintenance of intestinal homeostasis. Antibodies. 2013;2:535–553. doi: 10.3390/antib2040535.

[11] Akbar N, Khan NA, Muhammad JS, Siddiqui R. The role of gut microbiome in cancer genesis and cancer prevention. Health Sci Rev. 2022;2:100010. doi: 10.1016/j.hsr.2021.100010.

[12] Bertocchi A, Carloni S, Ravenda PS, Bertalot G, Spadoni I, Lo Cascio A, Gandini S, Lizier M, Braga D, Asnicar F, et al. Gut vascular barrier impairment leads to intestinal bacteria dissemination and colorectal cancer metastasis to liver. Cancer Cell. 2021;39:708–724 e711. doi: 10.1016/j.ccell.2021.03.004. 33798472

[13] Zheng D, Liwinski T, Elinav E. Interaction between microbiota and immunity in health and disease. Cell Res. 2020;30:492–506. doi: 10.1038/s41422-020-0332-7. 32433595 PMC7264227

[14] Kostic AD, Gevers D, Pedamallu CS, Michaud M, Duke F, Earl AM, Ojesina AI, Jung J, Bass AJ, Tabernero J, et al. Genomic analysis identifies association of *Fusobacterium* with colorectal carcinoma. Genome Res. 2012;22:292–298. doi: 10.1101/gr.126573.111. 22009990 PMC3266036

[15] Castellarin M, Warren RL, Freeman JD, Dreolini L, Krzywinski M, Strauss J, Barnes R, Watson P, Allen-Vercoe E, Moore RA, et al. *Fusobacterium nucleatum* infection is prevalent in human colorectal carcinoma. Genome Res. 2012;22:299–306. doi: 10.1101/gr.126516.111. 22009989 PMC3266037

[16] Abed J, Maalouf N, Manson AL, Earl AM, Parhi L, Emgård JEM, Klutstein M, Tayeb S, Almogy G, Atlan KA, et al. Colon cancer-associated Fusobacterium nucleatum may originate from the oral cavity and reach colon tumors via the circulatory system. Front Cell Infect Microbiol. 2020;10:400. doi: 10.3389/fcimb.2020.00400. 32850497 PMC7426652

[17] Komiya Y, Shimomura Y, Higurashi T, Sugi Y, Arimoto J, Umezawa S, Uchiyama S, Matsumoto M, Nakajima A. Patients with colorectal cancer have identical strains of *fusobacterium nucleatum* in their colorectal cancer and oral cavity. Gut. 2019;68:1335–1337. doi: 10.1136/gutjnl-2018-316661. 29934439 PMC6582823

[18] Morsy Y, Walberg [, Wawrzyniak P, Hubeli B, Truscello L, Mamie C, Niechcial A, Gueguen E, Manzini R, Gottier C, et al. Blood-borne immune cells carry low biomass DNA remnants of microbes in patients with colorectal cancer or inflammatory bowel disease. Gut Microbes. 2025;17:2530157. doi: 10.1080/19490976.2025.2530157. 40685618 PMC12283013

[19] Schorr L, Mathies M, Elinav E, Puschhof J. Intracellular bacteria in cancer—prospects and debates. NPJ Biofilms Microbiomes. 2023;9:76. doi: 10.1038/s41522-023-00446-9. 37813921 PMC10562400

[20] Montalban-Arques A, Katkeviciute E, Busenhart P, Bircher A, Wirbel J, Zeller G, Morsy Y, Borsig L, Glaus Garzon JF, Müller A, et al. Commensal clostridiales strains mediate effective anti-cancer immune response against solid tumors. Cell Host Microbe. 2021;29:1573–1588 e1577. doi: 10.1016/j.chom.2021.08.001. 34453895

[21] Wang Y, Zhang C, Hou S, Wu X, Liu J, Wan X. Analyses of potential driver and passenger bacteria in human colorectal cancer. CMAR. 2020;12:11553–11561. doi: 10.2147/CMAR.S275316. PMC7669530 33209059

[22] Nejman D, Livyatan I, Fuks G, Gavert N, Zwang Y, Geller LT, Rotter-Maskowitz A, Weiser R, Mallel G, Gigi E, et al. The human tumor microbiome is composed of tumor type-specific intracellular bacteria. Science. 2020;368:973–980. doi: 10.1126/science.aay9189. 32467386 PMC7757858

[23] Fu A, Yao B, Dong T, Chen Y, Yao J, Liu Y, Li H, Bai H, Liu X, Zhang Y, et al. Tumor-resident intracellular microbiota promotes metastatic colonization in breast cancer. Cell. 2022;185:1356–1372 e1326. doi: 10.1016/j.cell.2022.02.027. 35395179

[24] Kalaora S, Nagler A, Nejman D, Alon M, Barbolin C, Barnea E, Ketelaars SLC, Cheng K, Vervier K, Shental N, et al. Identification of bacteria-derived HLA-bound peptides in melanoma. Nature. 2021;592:138–143. doi: 10.1038/s41586-021-03368-8. 33731925 PMC9717498

[25] Bullman S, Pedamallu CS, Sicinska E, Clancy TE, Zhang X, Cai D, Neuberg D, Huang K, Guevara F, Nelson T, et al. Analysis of fusobacterium persistence and antibiotic response in colorectal cancer. Sci. 2017;358:1443–1448. doi: 10.1126/science.aal5240. PMC5823247 29170280

[26] Dejea CM, Wick EC, Hechenbleikner EM, White JR, Mark Welch JL, Rossetti BJ, Peterson SN, Snesrud EC, Borisy GG, Lazarev M, et al. Microbiota organization is a distinct feature of proximal colorectal cancers. Proc Natl Acad Sci USA. 2014;111:18321–18326. doi: 10.1073/pnas.1406199111. 25489084 PMC4280621

[27] Kvich L, Fritz BG, Zschach H, Terkelsen T, Raskov H, Høst-Rasmussen K, Jakobsen MR, Gheorghe AG, Gögenur I, Bjarnsholt T. Biofilms and core pathogens shape the tumor microenvironment and immune phenotype in colorectal cancer. Gut Microbes. 2024;16:2350156. doi: 10.1080/19490976.2024.2350156. 38726597 PMC11093030

[28] Dejea CM, Fathi P, Craig JM, Boleij A, Taddese R, Geis AL, Wu X, DeStefano Shields CE, Hechenbleikner EM, Huso DL, et al. Patients with familial adenomatous polyposis harbor colonic biofilms containing tumorigenic bacteria. Sci. 2018;359:592–597. doi: 10.1126/science.aah3648. PMC5881113 29420293

[29] Kolachala VL, Maddipatla SC, Murthy S, Hwang Y, Dodd AF, Sharma G, Munasinghe S, Pelia RS, Venkateswaran S, Anbazhagan M, et al. Altered inflammatory mucosal signatures within their spatial and cellular context during active ileal Crohn's disease. JCI Insight. 2025;10. doi: 10.1172/jci.insight.171783. PMC11949056 40059828

[30] Fabriek BO, Dijkstra CD, Van Den Berg TK. The macrophage scavenger receptor CD163. Immunobiology. 2005;210:153–160. doi: 10.1016/j.imbio.2005.05.010. 16164022

[31] Liang JQ, Li T, Nakatsu G, Chen Y-X, Yau TO, Chu E, Wong S, Szeto CH, Ng SC, Chan FKL, et al. A novel faecal Lachnoclostridium marker for the non-invasive diagnosis of colorectal adenoma and cancer. Gut *69*, 1248. Gut. 2020;69:1248–1257. doi: 10.1136/gutjnl-2019-318532. 31776231 PMC7306980

[32] Li Q, Xiao Y, Han L, Luo W, Dai W, Fang H, Wang R, Xu Y, Cai S, Goel A, et al. Microbiome dysbiosis, neutrophil recruitment and mesenchymal transition of mesothelial cells promotes peritoneal metastasis of colorectal cancer. Nat Cancer. 2025;6:493–510. doi: 10.1038/s43018-025-00910-9. 39966610

[33] Galeano Niño JL, Ponath F, Ajisafe VA, Becker CR, Kempchinsky AG, Zepeda-Rivera MA, Gomez JA, Wu H, Terrazas JG, Bouzek H, et al. Tumor-infiltrating bacteria disrupt cancer epithelial cell interactions and induce cell-cycle arrest. Cancer Cell. 2026;44:166–186 e116. doi: 10.1016/j.ccell.2025.09.010. 41106380 PMC12774452

[34] Silva LM, Kim TS, Moutsopoulos NM. Neutrophils are gatekeepers of mucosal immunity. Immunol Rev. 2023;314:125–141. doi: 10.1111/imr.13171. 36404627 PMC10496120

[35] Zhang D, Frenette PS. Cross talk between neutrophils and the microbiota. Blood. 2019;133:2168–2177. doi: 10.1182/blood-2018-11-844555. 30898860 PMC6524562

[36] Fischer JC, Wintges A, Haas T, Poeck H. Assessment of mucosal integrity by quantifying neutrophil granulocyte influx in murine models of acute intestinal injury. Cell Immunol. 2017;316:70–76. doi: 10.1016/j.cellimm.2017.04.003. 28413062

[37] Martin A, Seignez C, Racoeur C, Isambert N, Mabrouk N, Scagliarini A, Reveneau S, Arnould L, Bettaieb A, Jeannin J-F, et al. Tumor-derived granzyme B-expressing neutrophils acquire antitumor potential after lipid A treatment. Oncotarget. 2018;9:28364–28378. doi: 10.18632/oncotarget.25342. 29983866 PMC6033356

[38] Shen P, Cheng P, Li Y, Zong G, Deng R, Qian C, Zhao Y, Wei Z, Lu Y. Unveiling the covert interaction between gut microbiota and neutrophils to drive colorectal cancer metastasis. Eur J Pharmacol. 2024;962:176217. doi: 10.1016/j.ejphar.2023.176217. 38036200

[39] Segal BH, Giridharan T, Suzuki S, Khan ANH, Zsiros E, Emmons TR, Yaffe MB, Gankema AAF, Hoogeboom M, Goetschalckx I, et al. Neutrophil interactions with T cells, platelets, endothelial cells, and of course tumor cells. Immunol Rev. 2023;314:13–35. doi: 10.1111/imr.13178. 36527200 PMC10174640

[40] Germann M, Zangger N, Sauvain MO, Sempoux C, Bowler AD, Wirapati P, Kandalaft LE, Delorenzi M, Tejpar S, Coukos G, et al. Neutrophils suppress tumor-infiltrating T cells in colon cancer via matrix metalloproteinase-mediated activation of. TGFbeta. EMBO Mol Med. 2020;12:e10681. doi: 10.15252/emmm.201910681. 31793740 PMC6949488

[41] Oberg HH, Wesch D, Kalyan S, Kabelitz D. Regulatory interactions between neutrophils, tumor cells and T cells. Front Immunol. 2019;10:1690. doi: 10.3389/fimmu.2019.01690. 31379875 PMC6657370

[42] Zhu M, Jia R, Zhang X, Xu P. The success of the tumor immunotherapy: neutrophils from bench to beside. Front Immunol. 2025;16:1524038. doi: 10.3389/fimmu.2025.1524038. 39925807 PMC11802522

[43] Mysore V, Cullere X, Mears J, Rosetti F, Okubo K, Liew PX, Zhang F, Madera-Salcedo I, Rosenbauer F, Stone RM, et al. FcγR engagement reprograms neutrophils into antigen cross-presenting cells that elicit acquired anti-tumor immunity. Nat Commun. 2021;12:4791. doi: 10.1038/s41467-021-24591-x. 34373452 PMC8352912

