# Supplementary material for: Altered crosstalk of bacterial lipopolysaccharide with immune cells in colorectal cancer compared to paired adjacent intestinal tissue
Source: Gut Microbes. 2026 May 5;18(1):2665878. doi: 10.1080/19490976.2026.2665878 (PMC13154938; doi:10.1080/19490976.2026.2665878)
Supplement: Supplementary Material — Figures_Supplement_reduced.pdf [file KGMI_A_2665878_SM3617.pdf]

S1

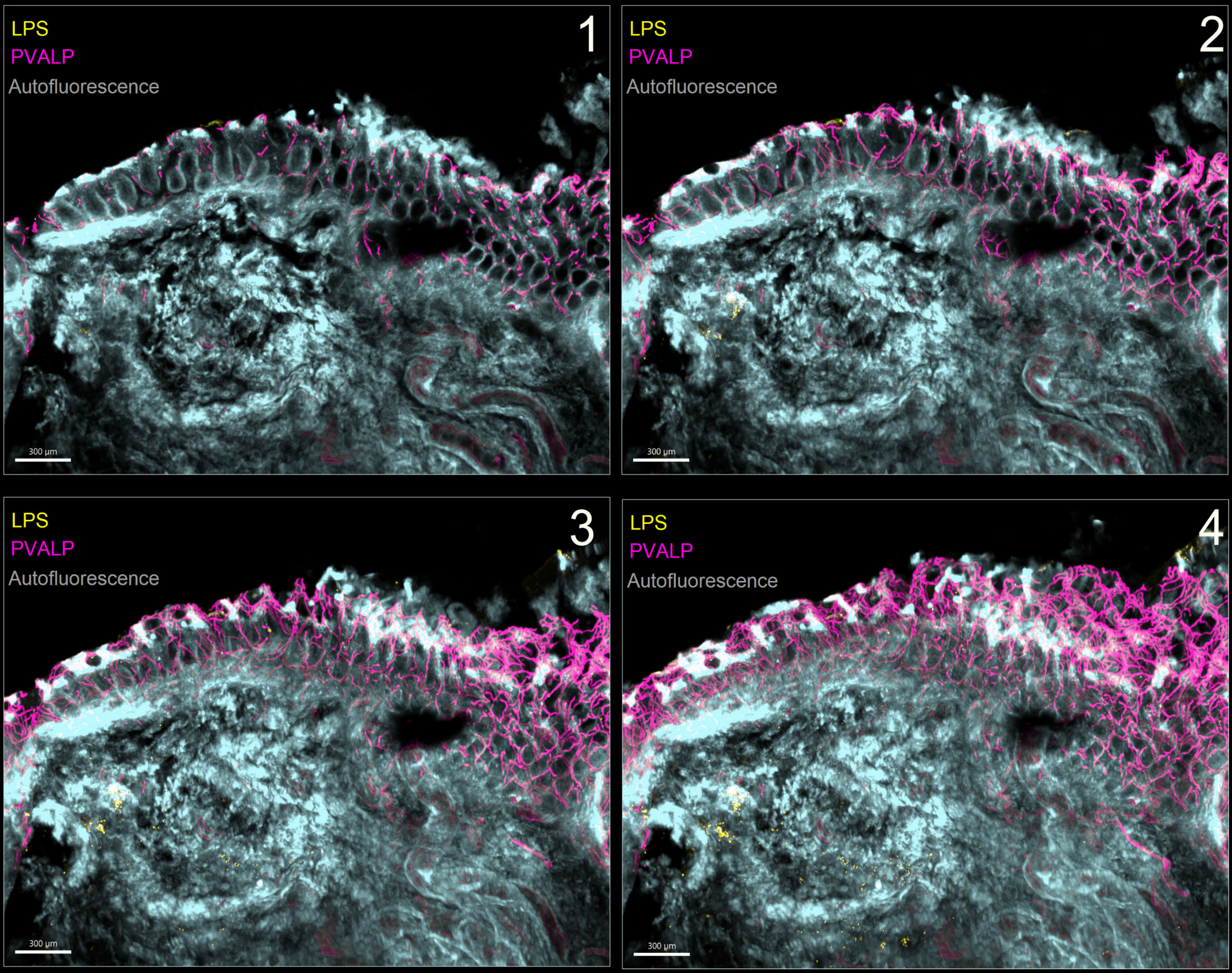
LPS
PVALP
Autofluorescence
1
300 µm
LPS
PVALP
Autofluorescence
2
300 µm
LPS
PVALP
Autofluorescence
3
300 µm
LPS
PVALP
Autofluorescence
4
300 µm

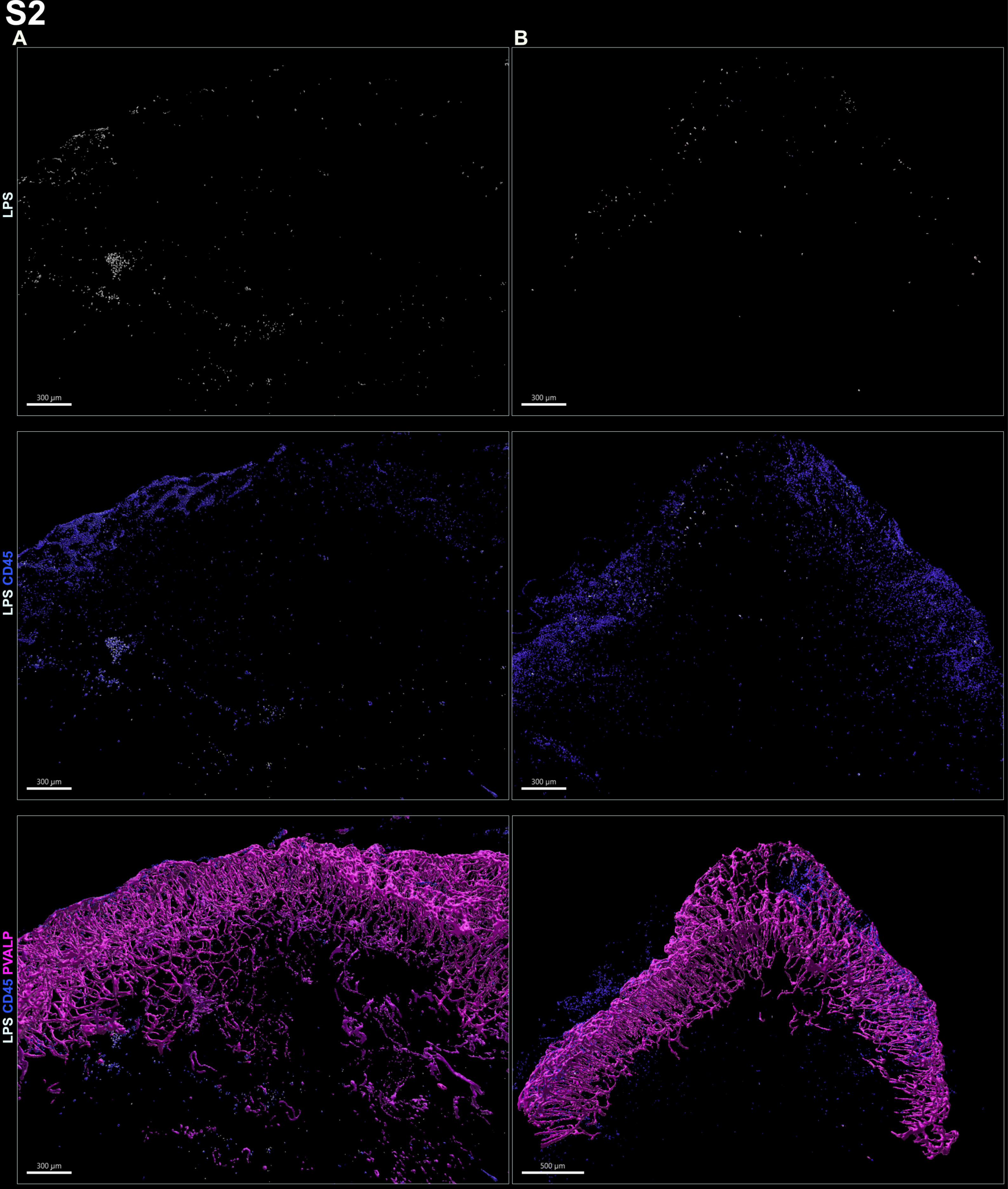
S2
A
B
LPS
LPS CD45
LPS CD45 PVALP
300 µm
500 µm

S3

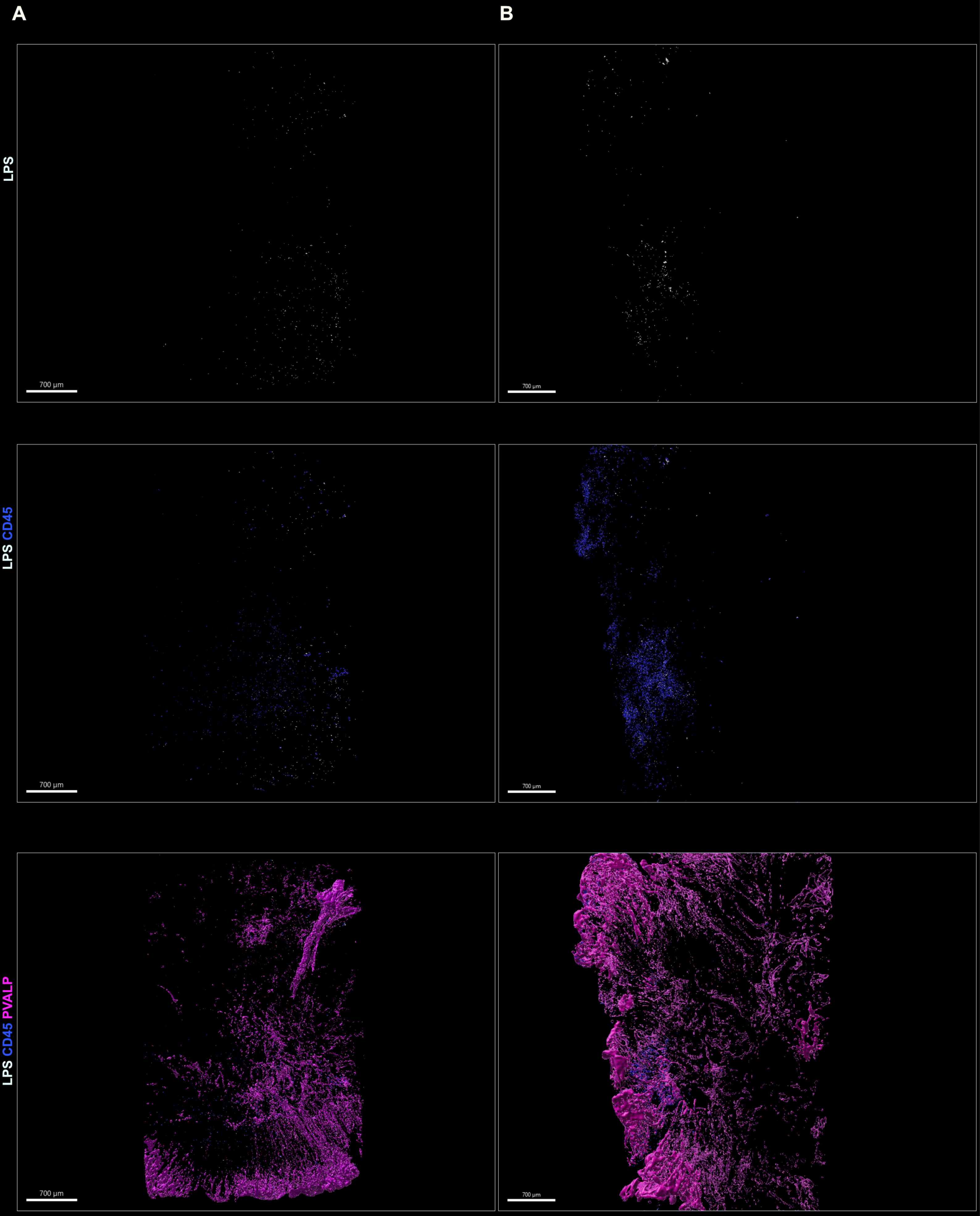
A
B
LPS
LPS CD45
LPS CD45 PVALP
700 µm
700 µm
700 µm
700 µm
700 µm
700 µm

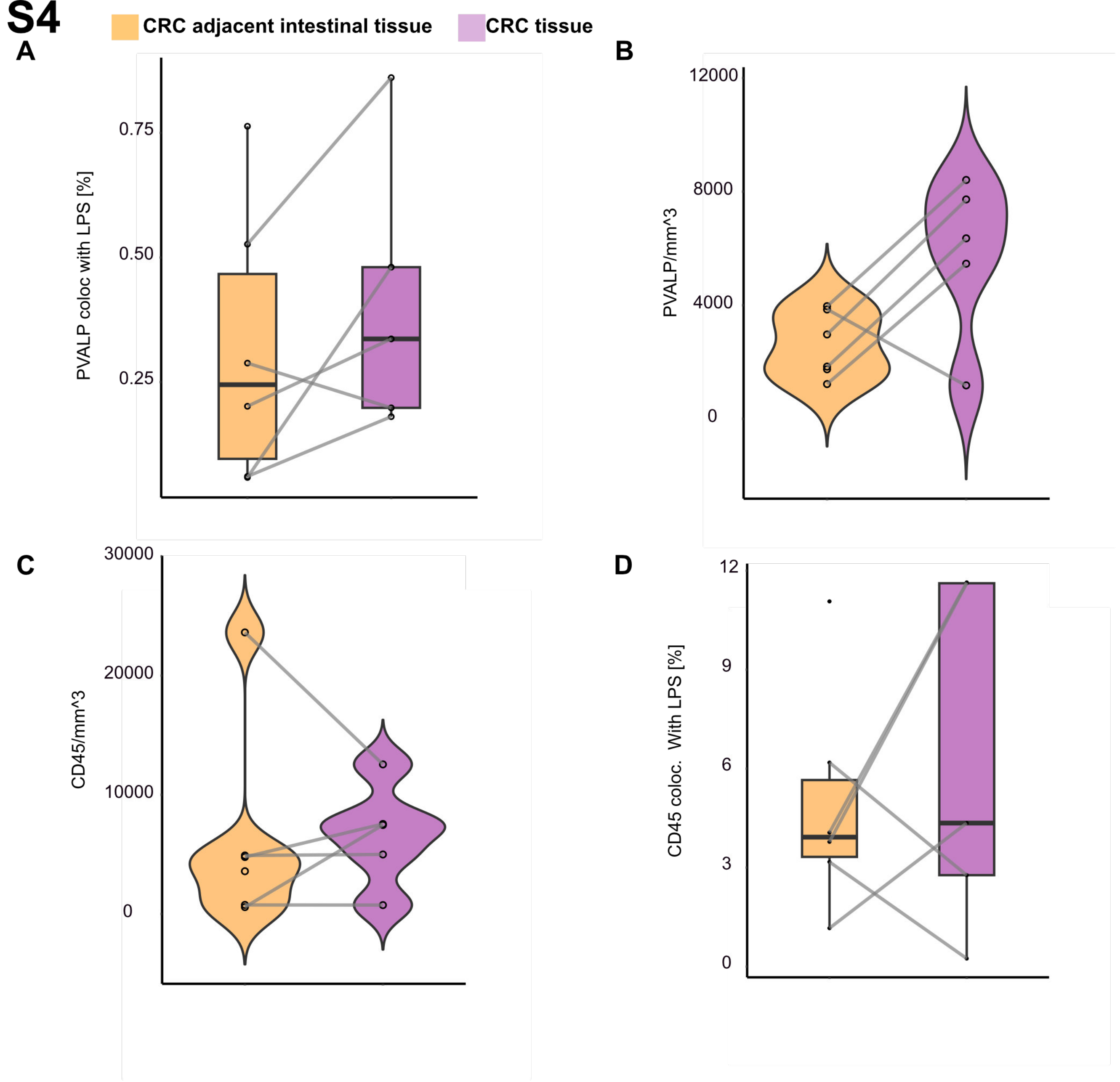

S4
CRC adjacent intestinal tissue
CRC tissue
A
PVALP coloc with LPS [%]
0.75
0.50
0.25
B
PVALP/mm^3
12000
8000
4000
0
C
CD45/mm^3
30000
20000
10000
0
D
CD45 coloc. With LPS [%]
12
9
6
3
0

S5

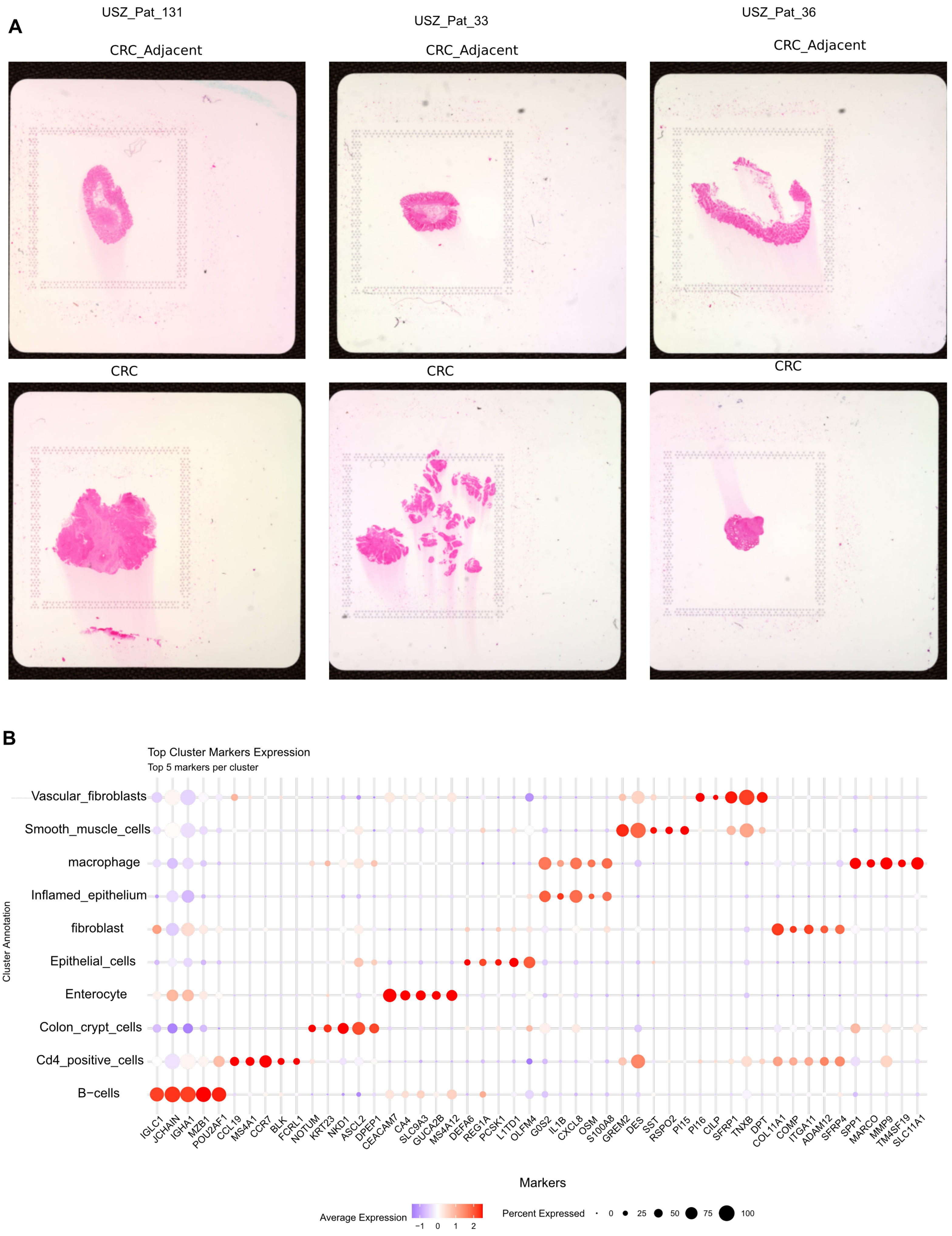

A
USZ_Pat_131
USZ_Pat_33
USZ_Pat_36
CRC_Adjacent
CRC_Adjacent
CRC_Adjacent
CRC
CRC
CRC
B
Top Cluster Markers Expression
Top 5 markers per cluster
Cluster Annotation
Vascular_fibroblasts
Smooth_muscle_cells
macrophage
Inflamed_epithelium
fibroblast
Epithelial_cells
Enterocyte
Colon_crypt_cells
Cd4_positive_cells
B−cells
IGLC1 JCHAIN IGHA1 MZB1 POU2AF1 CCL19 MS4A1 CCR7 BLK FCRL1 NOTUM KRT23 NKD1 ASCL2 DPEP1 CEACAM7 CA4 SLC9A3 GUCA2B MS4A12 DEFA6 REG1A PCSK1 L1TD1 OLFM4 G0S2 IL1B CXCL8 OSM S100A8 GREM2 DES SST RSPO2 PI15 PI16 CILP SFRP1 TNXB DPT COL11A1 COMP ITGA11 ADAM12 SFRP4 SPP1 MARCO MMP9 TM4SF19 SLC11A1
Markers
Average Expression
−1 0 1 2
Percent Expressed
0 25 50 75 100

S6

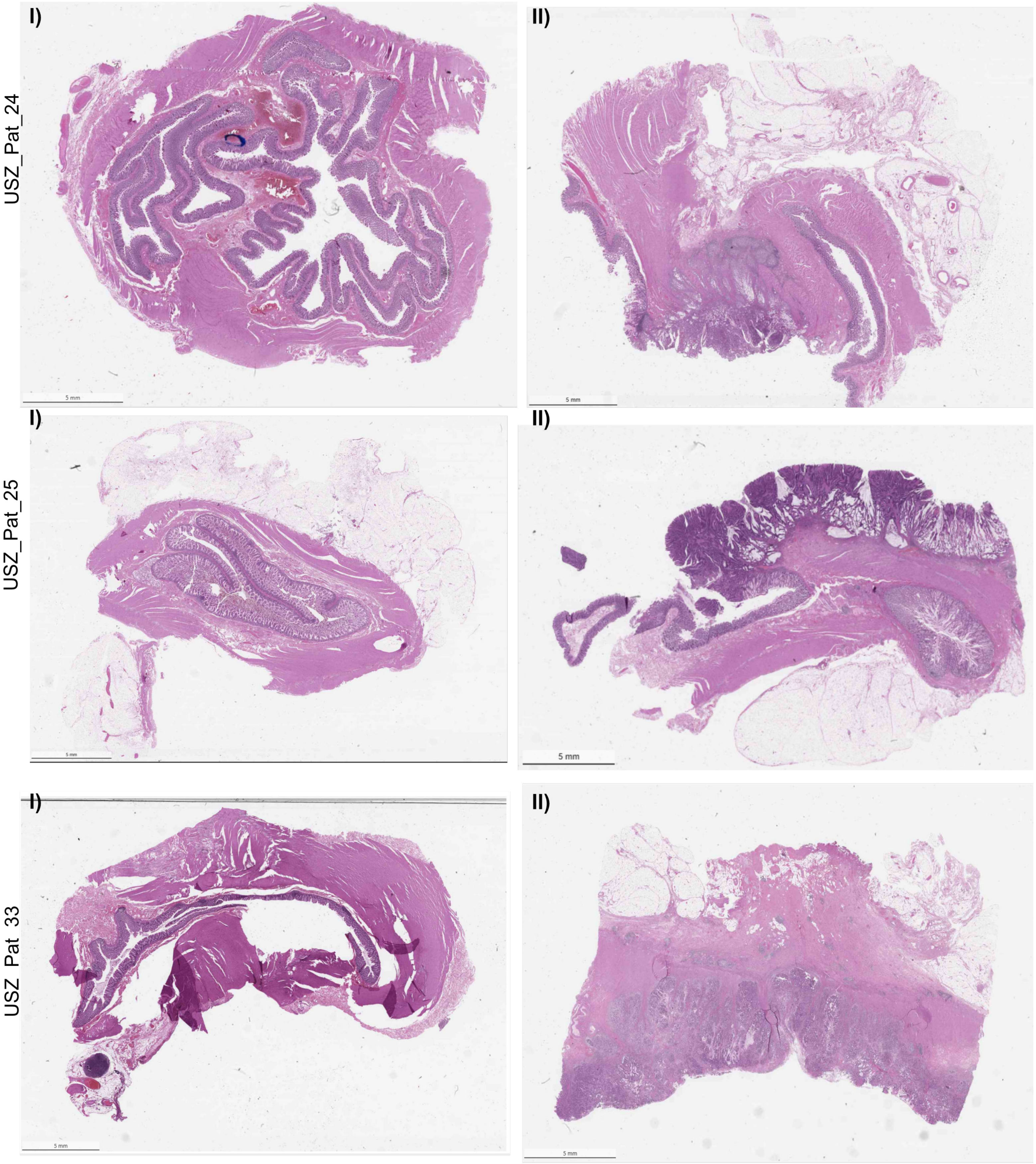

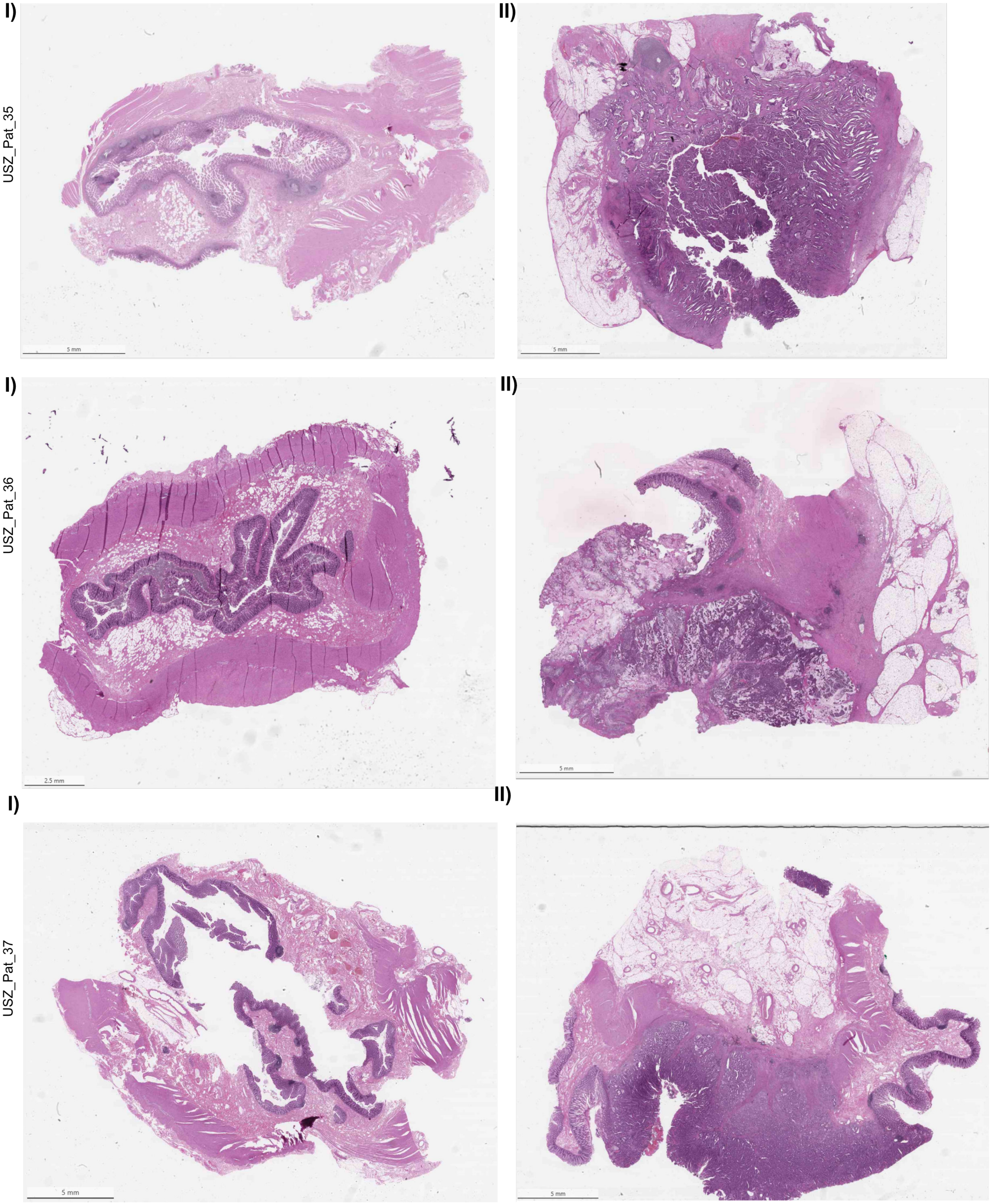

USZ_Pat_35
I)
5 mm
II)
5 mm
USZ_Pat_36
I)
2.5 mm
II)
5 mm
USZ_Pat_37
I)
5 mm
II)
5 mm

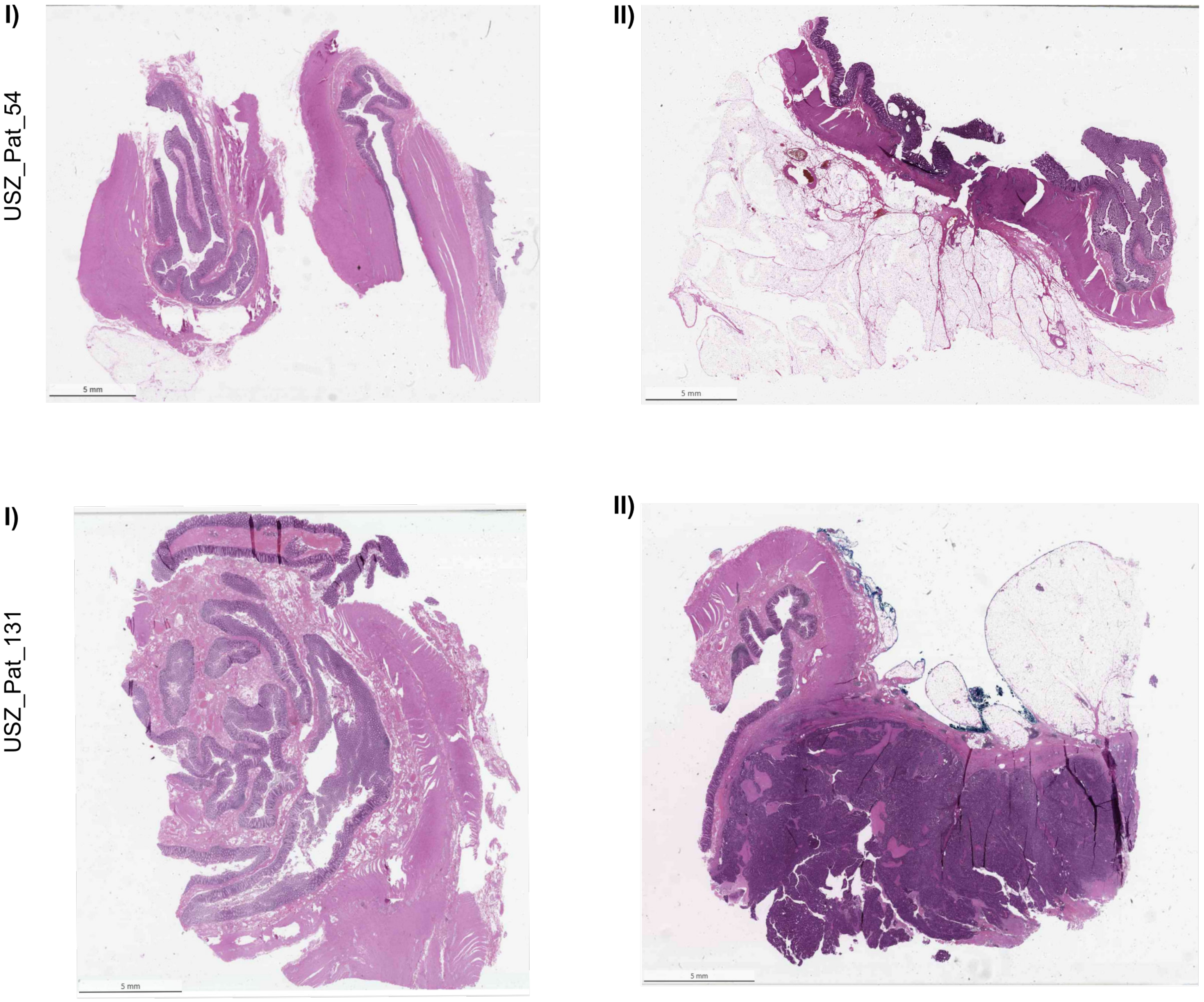
I)
II)
USZ_Pat_54
5 mm
5 mm
I)
II)
USZ_Pat_131
5 mm
5 mm

S7

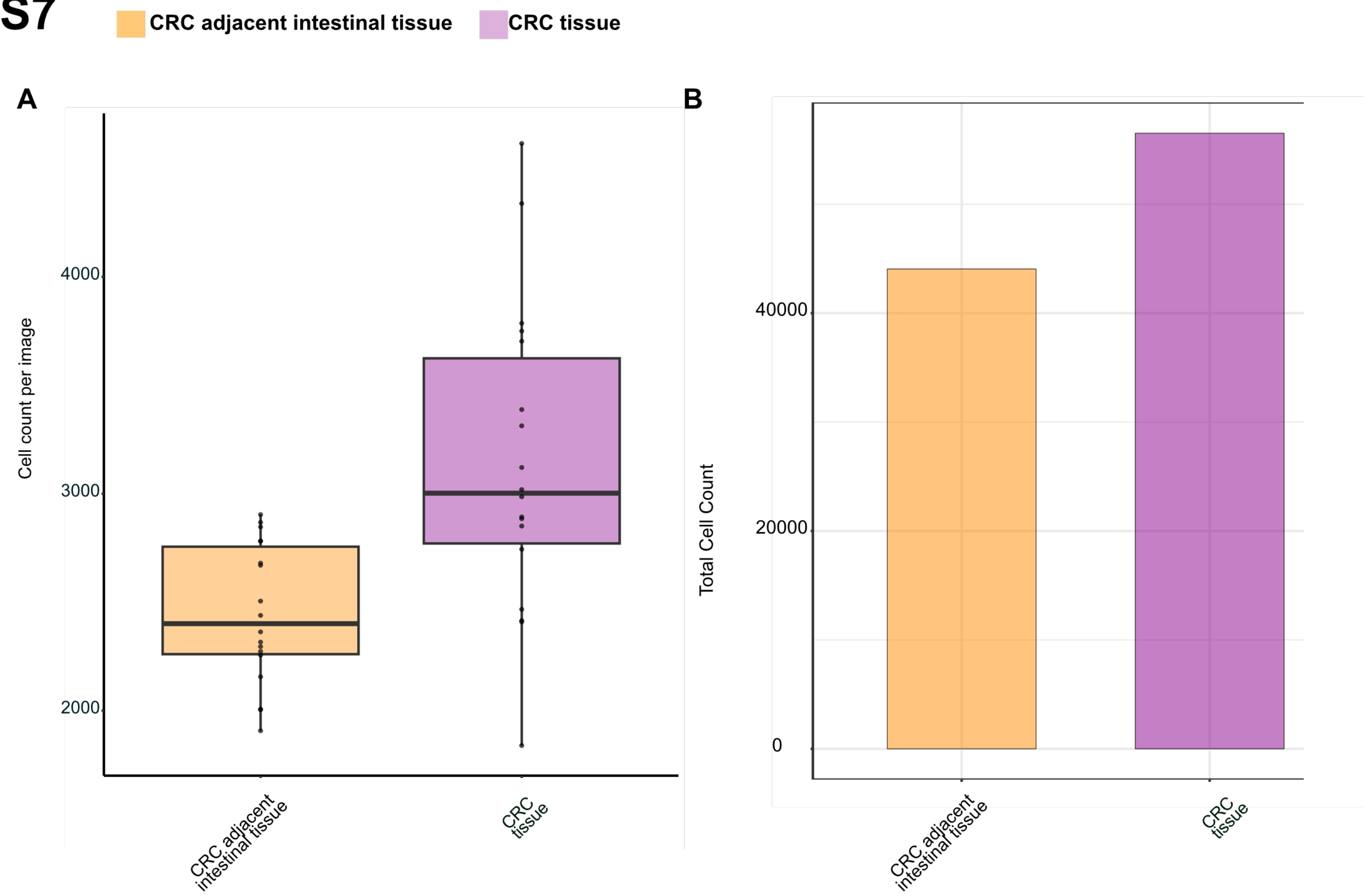

CRC adjacent intestinal tissue
CRC tissue
A
Cell count per image
4000
3000
2000
CRC adjacent intestinal tissue
CRC tissue
B
Total Cell Count
40000
20000
0
CRC adjacent intestinal tissue
CRC tissue

S8

A

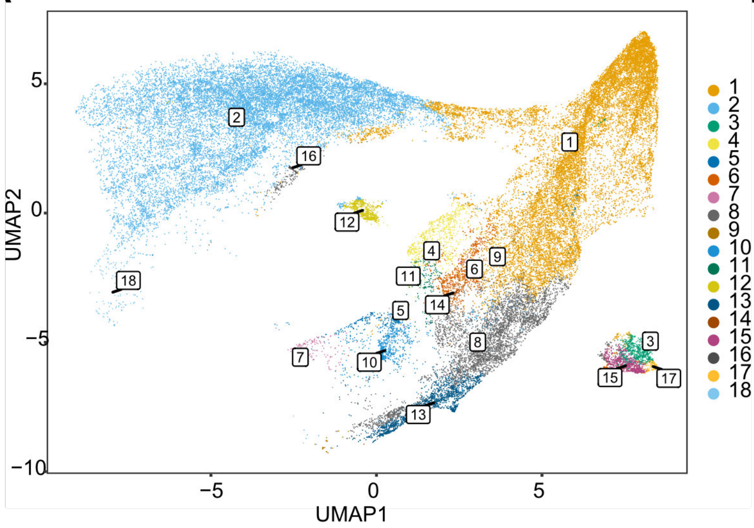
UMAP1
UMAP2
1
2
3
4
5
6
7
8
9
10
11
12
13
14
15
16
17
18

B

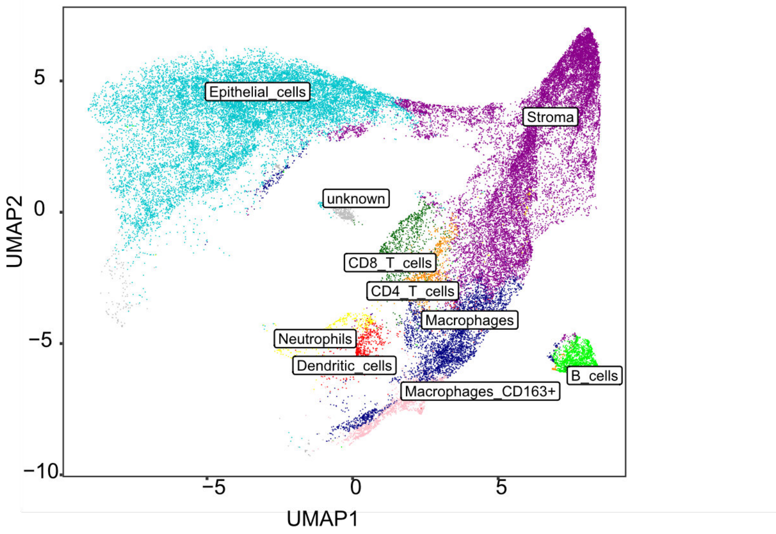
Epithelial_cells
Stroma
unknown
CD8_T_cells
CD4_T_cells
Macrophages
Neutrophils
Dendritic_cells
Macrophages_CD163+
B_cells
UMAP1
UMAP2

C

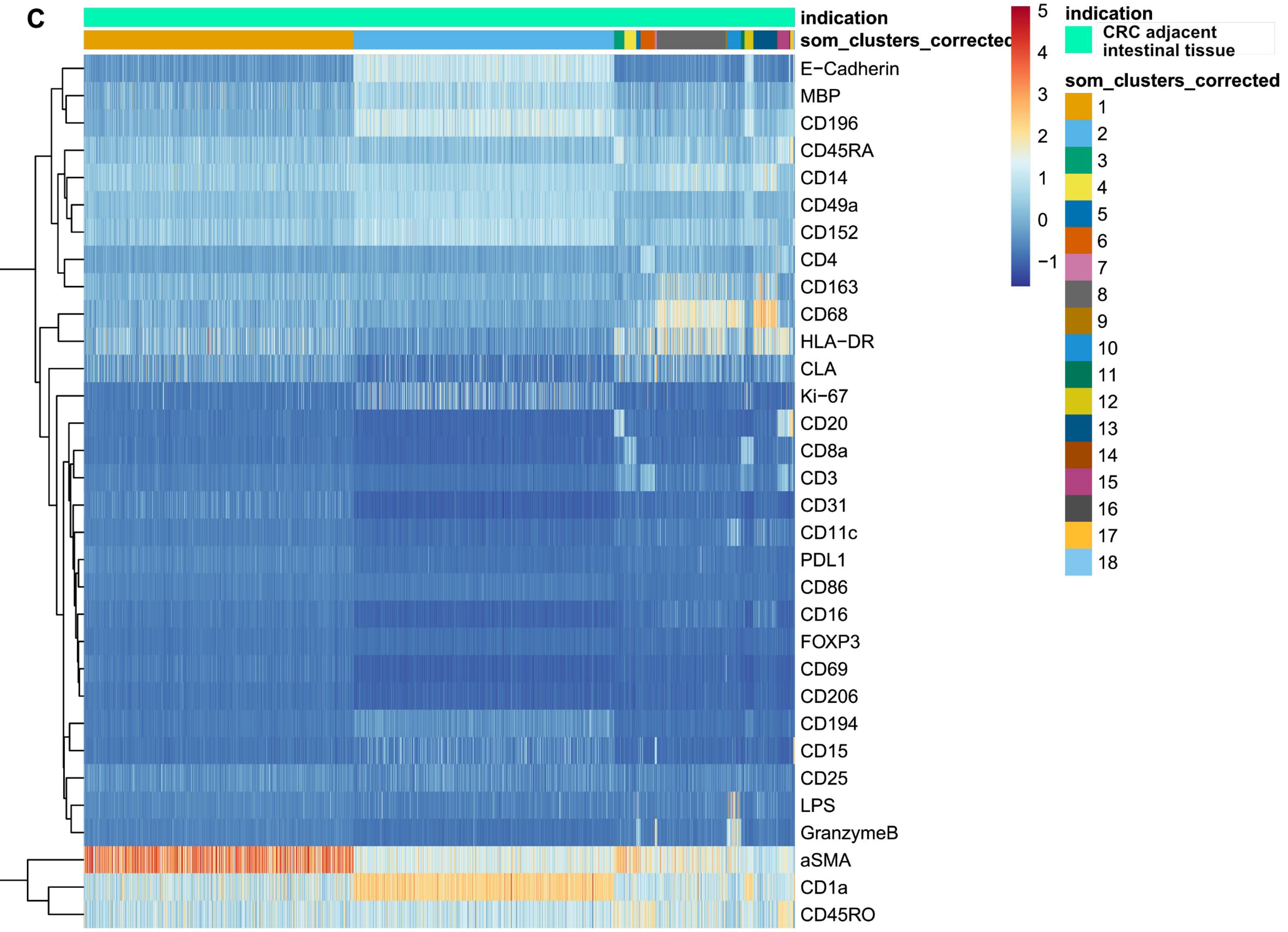
indication
som_clusters_corrected
E−Cadherin
MBP
CD196
CD45RA
CD14
CD49a
CD152
CD4
CD163
CD68
HLA−DR
CLA
Ki−67
CD20
CD8a
CD3
CD31
CD11c
PDL1
CD86
CD16
FOXP3
CD69
CD206
CD194
CD15
CD25
LPS
GranzymeB
aSMA
CD1a
CD45RO
indication
CRC adjacent intestinal tissue
som_clusters_corrected

S9

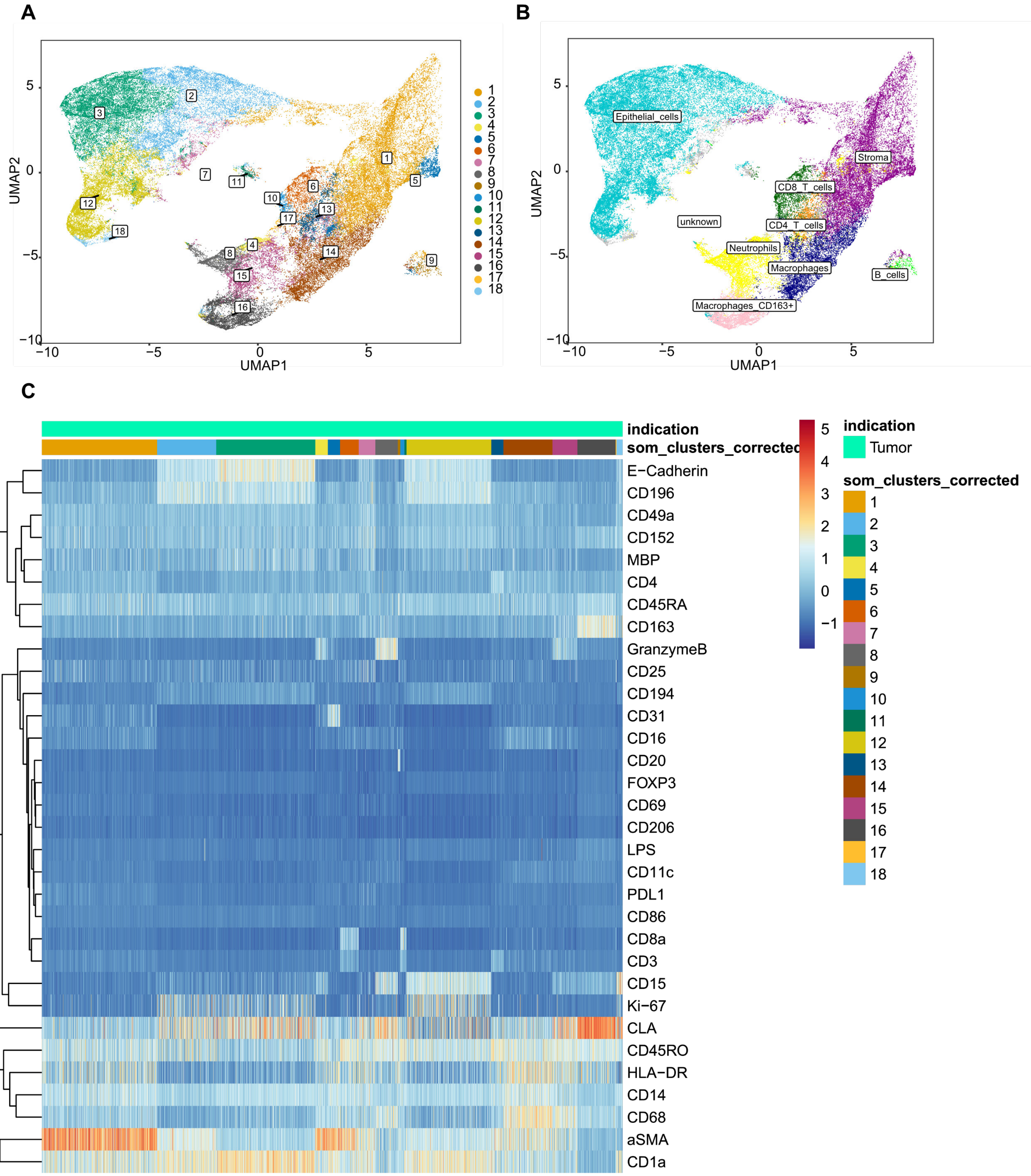
A
B
C
UMAP1
UMAP2
Epithelial_cells
Stroma
CD8_T_cells
CD4_T_cells
unknown
Neutrophils
Macrophages
Macrophages_CD163+
B_cells
indication
som_clusters_corrected
Tumor
E−Cadherin
CD196
CD49a
CD152
MBP
CD4
CD45RA
CD163
GranzymeB
CD25
CD194
CD31
CD16
CD20
FOXP3
CD69
CD206
LPS
CD11c
PDL1
CD86
CD8a
CD3
CD15
Ki−67
CLA
CD45RO
HLA−DR
CD14
CD68
aSMA
CD1a

S10

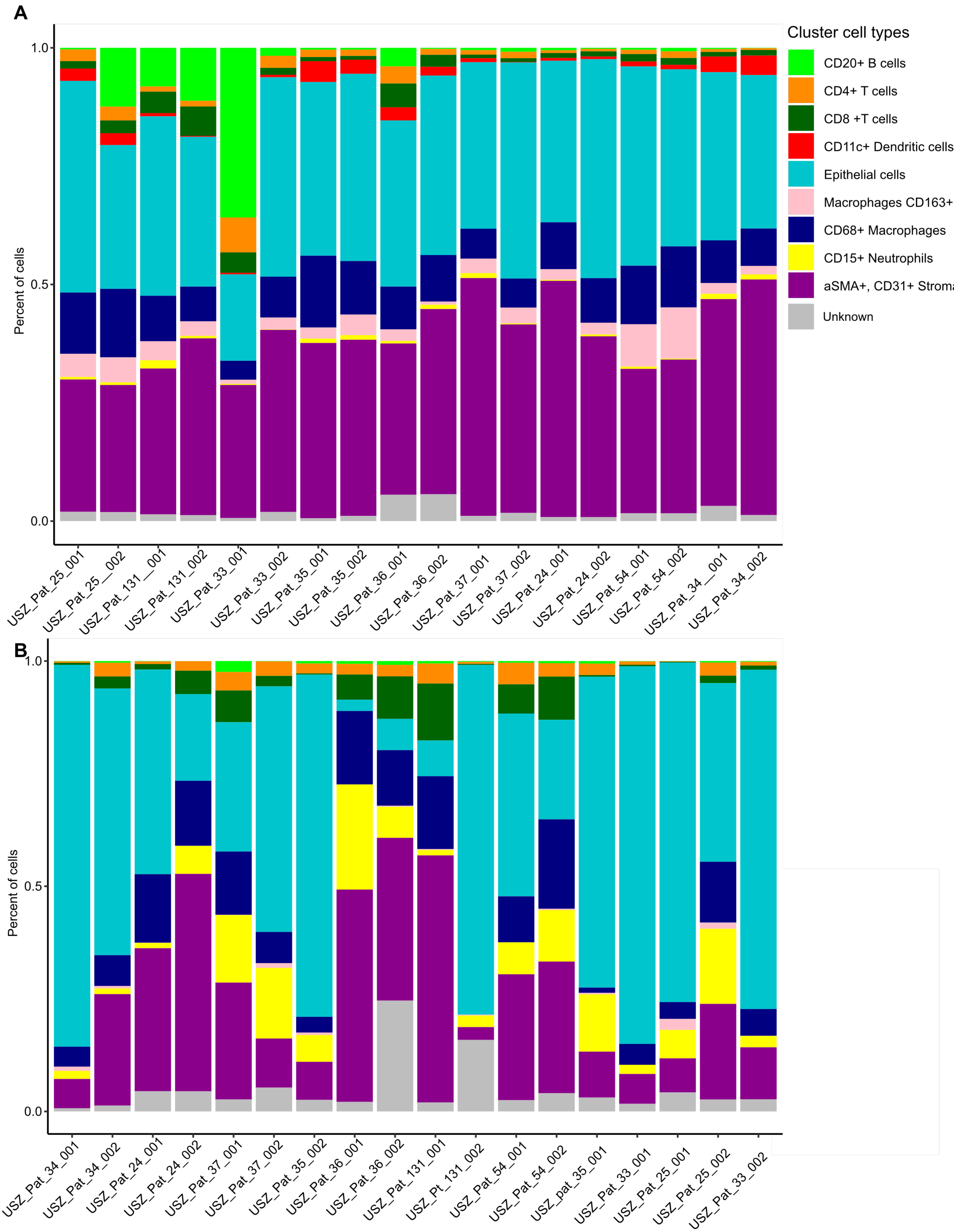
A
Percent of cells
1.0
0.5
0.0
Cluster cell types
CD20+ B cells
CD4+ T cells
CD8 +T cells
CD11c+ Dendritic cells
Epithelial cells
Macrophages CD163+
CD68+ Macrophages
CD15+ Neutrophils
aSMA+, CD31+ Stroma
Unknown
USZ_Pat_25_001
USZ_Pat_25 _002
USZ_Pat_131 _001
USZ_Pat_131_002
USZ_Pat_33_001
USZ_Pat_33_002
USZ_Pat_35_001
USZ_Pat_35_002
USZ_Pat_36_001
USZ_Pat_36_002
USZ_Pat_37_001
USZ_Pat_37_002
USZ_Pat_24_001
USZ_Pat_24_002
USZ_Pat_54_001
USZ_Pat_54_002
USZ_Pat_34 _001
USZ_Pat_34 _002
B
Percent of cells
1.0
0.5
0.0
USZ_Pat_34_001
USZ_Pat_34_002
USZ_Pat_24_001
USZ_Pat_24_002
USZ_Pat_37_001
USZ_Pat_37_002
USZ_Pat_35_002
USZ_Pat_36_001
USZ_Pat_36_002
USZ_Pat_131_001
USZ_Pt_131_002
USZ_Pat_54_001
USZ_Pat_54_002
USZ_pat_35_001
USZ_Pat_33_001
USZ_Pat_25_001
USZ_Pat_25_002
USZ_Pat_33_002

S11

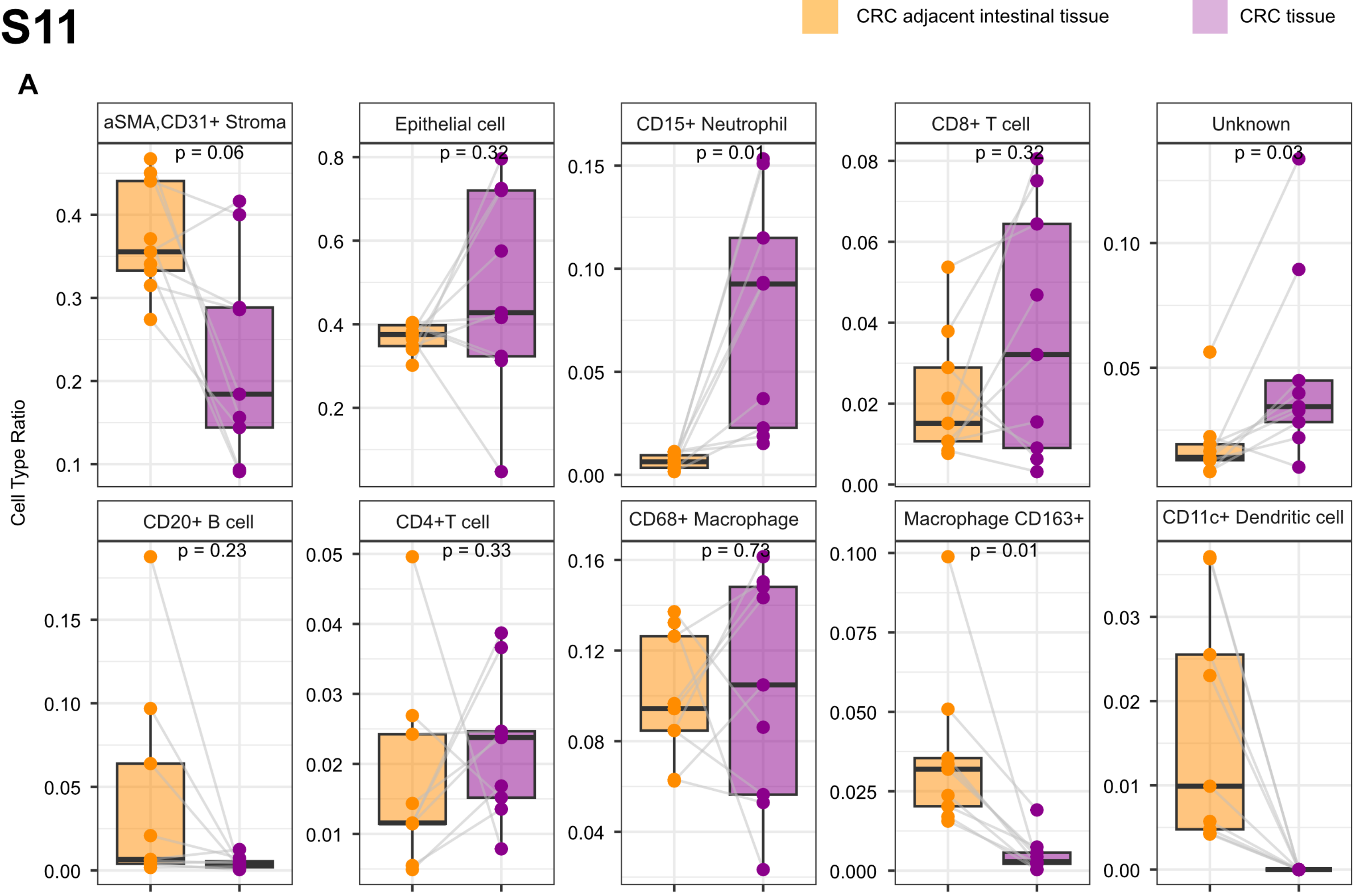
CRC adjacent intestinal tissue
CRC tissue
A
Cell Type Ratio
aSMA,CD31+ Stroma
p = 0.06
Epithelial cell
p = 0.32
CD15+ Neutrophil
p = 0.01
CD8+ T cell
p = 0.32
Unknown
p = 0.03
CD20+ B cell
p = 0.23
CD4+T cell
p = 0.33
CD68+ Macrophage
p = 0.73
Macrophage CD163+
p = 0.01
CD11c+ Dendritic cell

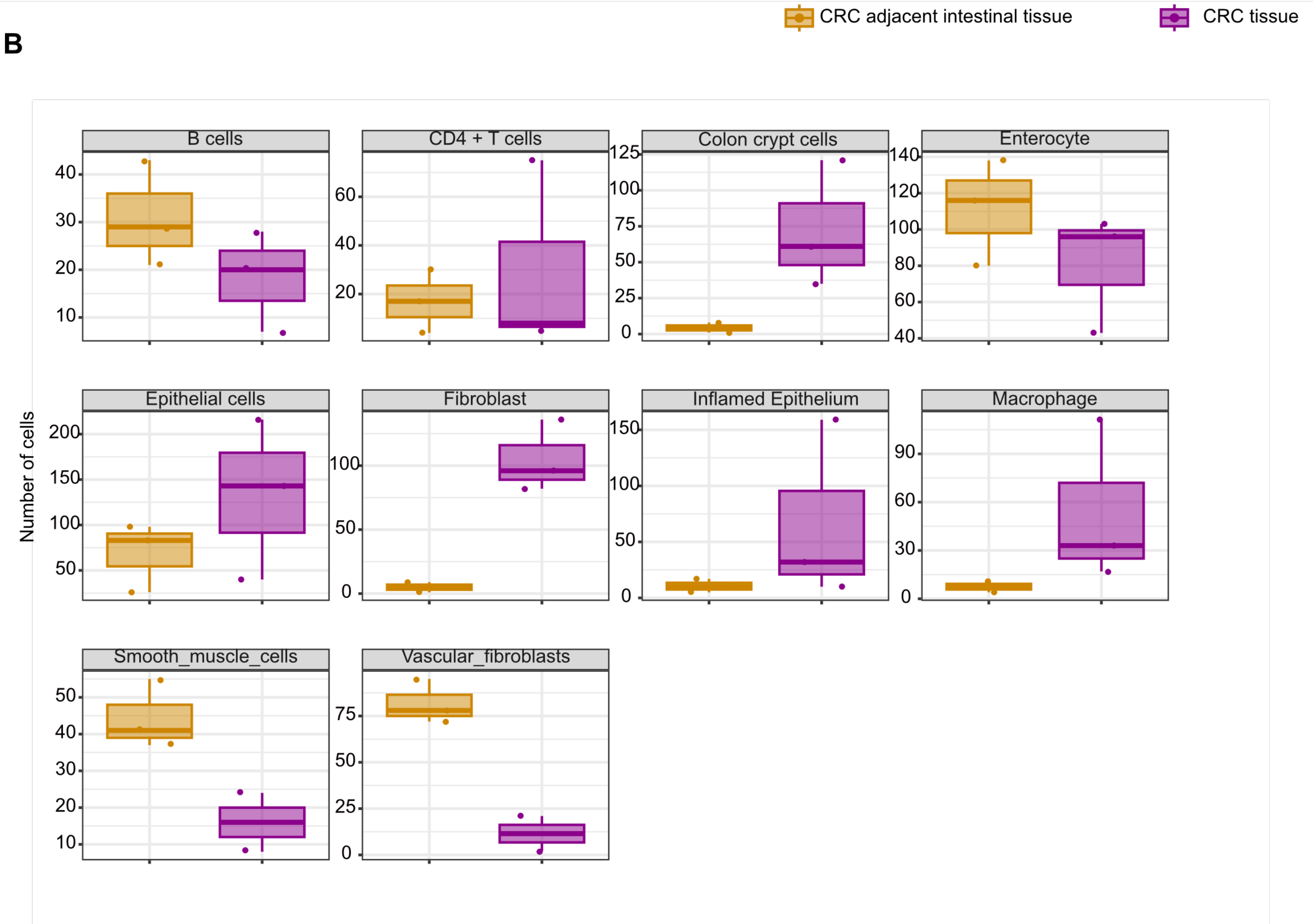
CRC adjacent intestinal tissue
CRC tissue
B
Number of cells
B cells
CD4 + T cells
Colon crypt cells
Enterocyte
Epithelial cells
Fibroblast
Inflamed Epithelium
Macrophage
Smooth_muscle_cells
Vascular_fibroblasts

S12

A

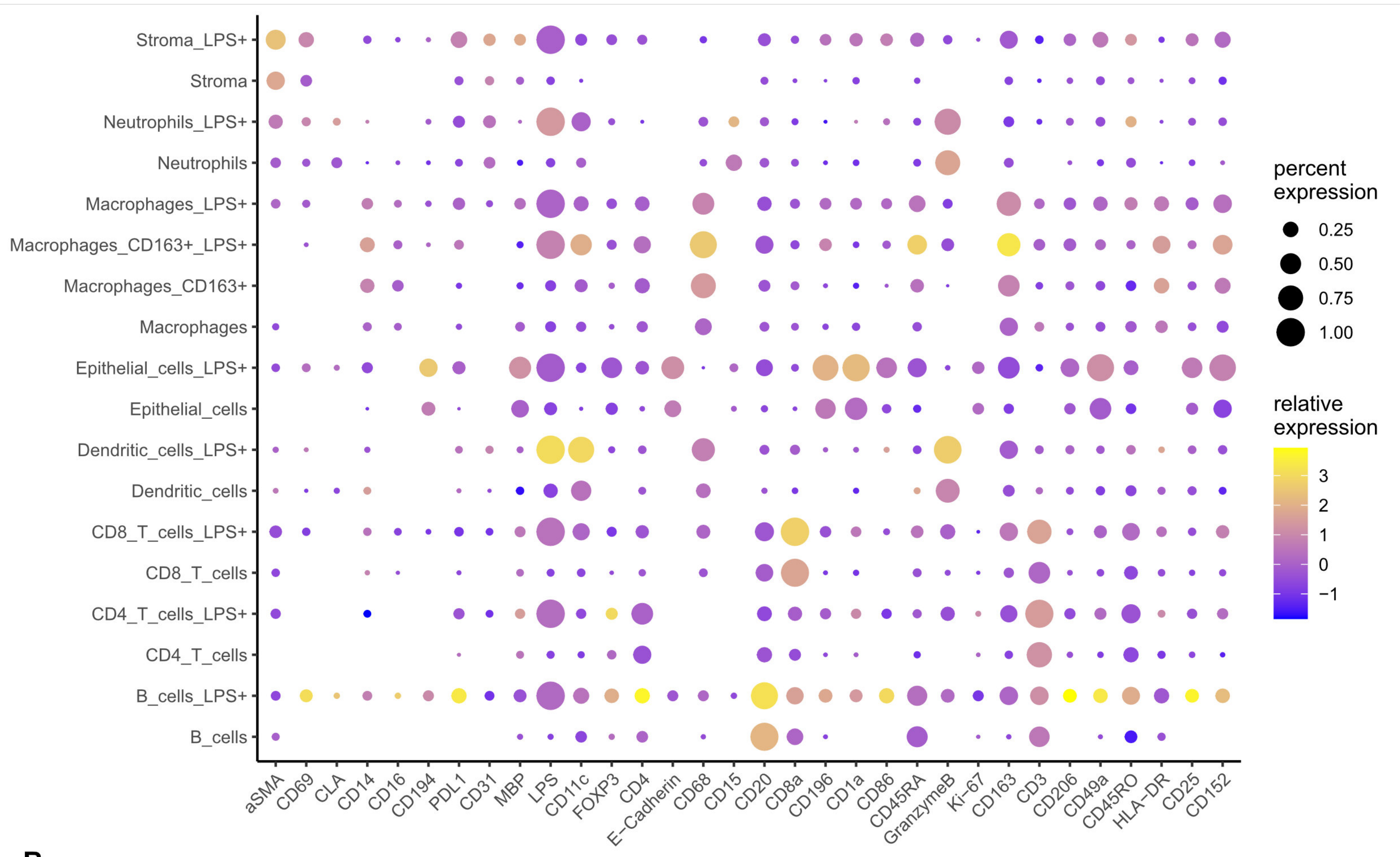
Stroma_LPS+
Stroma
Neutrophils_LPS+
Neutrophils
Macrophages_LPS+
Macrophages_CD163+_LPS+
Macrophages_CD163+
Macrophages
Epithelial_cells_LPS+
Epithelial_cells
Dendritic_cells_LPS+
Dendritic_cells
CD8_T_cells_LPS+
CD8_T_cells
CD4_T_cells_LPS+
CD4_T_cells
B_cells_LPS+
B_cells
aSMA CD69 CLA CD14 CD16 CD194 PDL1 CD31 MBP LPS CD11c FOXP3 CD4 E-Cadherin CD68 CD15 CD20 CD8a CD196 CD1a CD86 CD45RA GranzymeB Ki-67 CD163 CD3 CD206 CD49a CD45RO HLA-DR CD25 CD152
percent expression
0.25
0.50
0.75
1.00
relative expression
3
2
1
0
−1

B

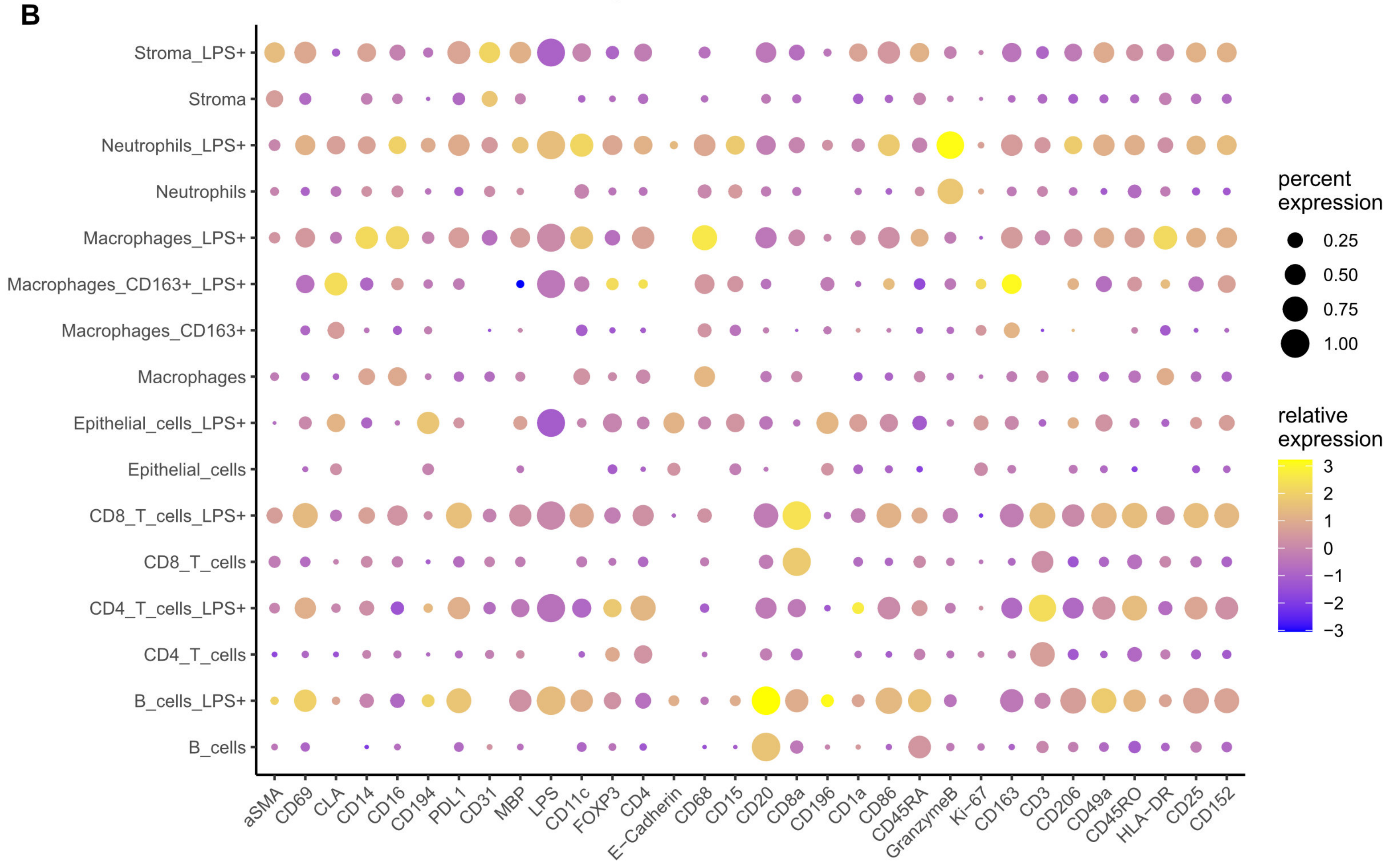
Stroma_LPS+
Stroma
Neutrophils_LPS+
Neutrophils
Macrophages_LPS+
Macrophages_CD163+_LPS+
Macrophages_CD163+
Macrophages
Epithelial_cells_LPS+
Epithelial_cells
CD8_T_cells_LPS+
CD8_T_cells
CD4_T_cells_LPS+
CD4_T_cells
B_cells_LPS+
B_cells
aSMA CD69 CLA CD14 CD16 CD194 PDL1 CD31 MBP LPS CD11c FOXP3 CD4 E-Cadherin CD68 CD15 CD20 CD8a CD196 CD1a CD86 CD45RA GranzymeB Ki-67 CD163 CD3 CD206 CD49a CD45RO HLA-DR CD25 CD152
percent expression
0.25
0.50
0.75
1.00
relative expression
3
2
1
0
−1
−2
−3

S13

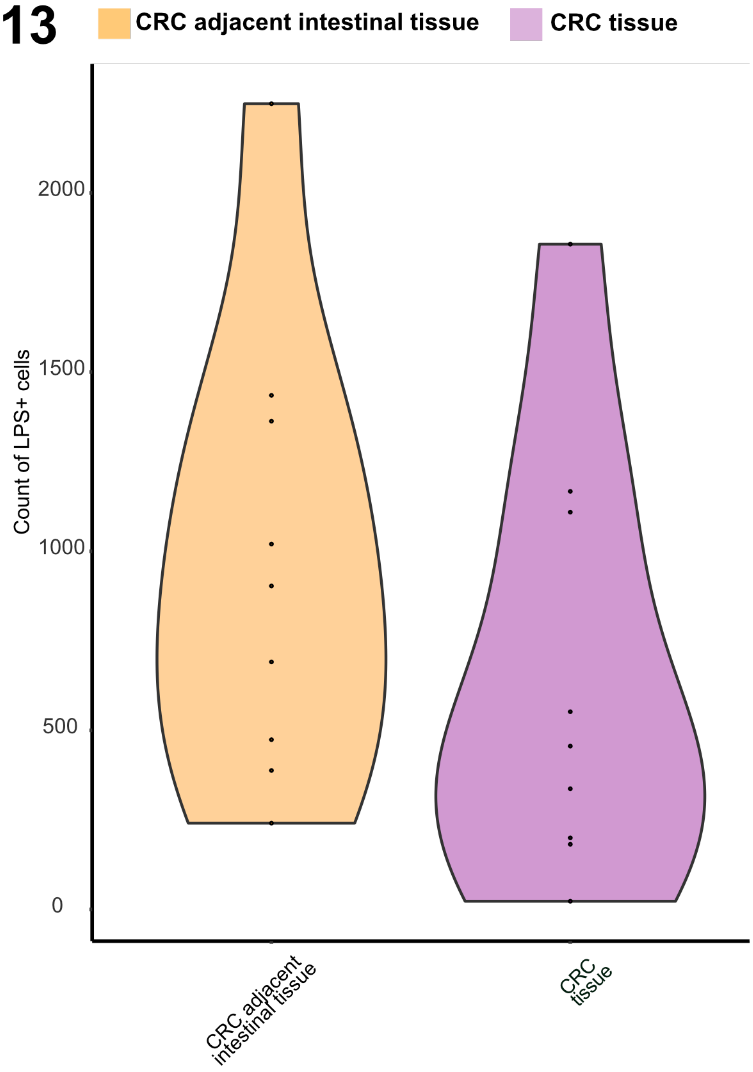

CRC adjacent intestinal tissue
CRC tissue
Count of LPS+ cells
2000
1500
1000
500
0
CRC adjacent intestinal tissue
CRC tissue

S14

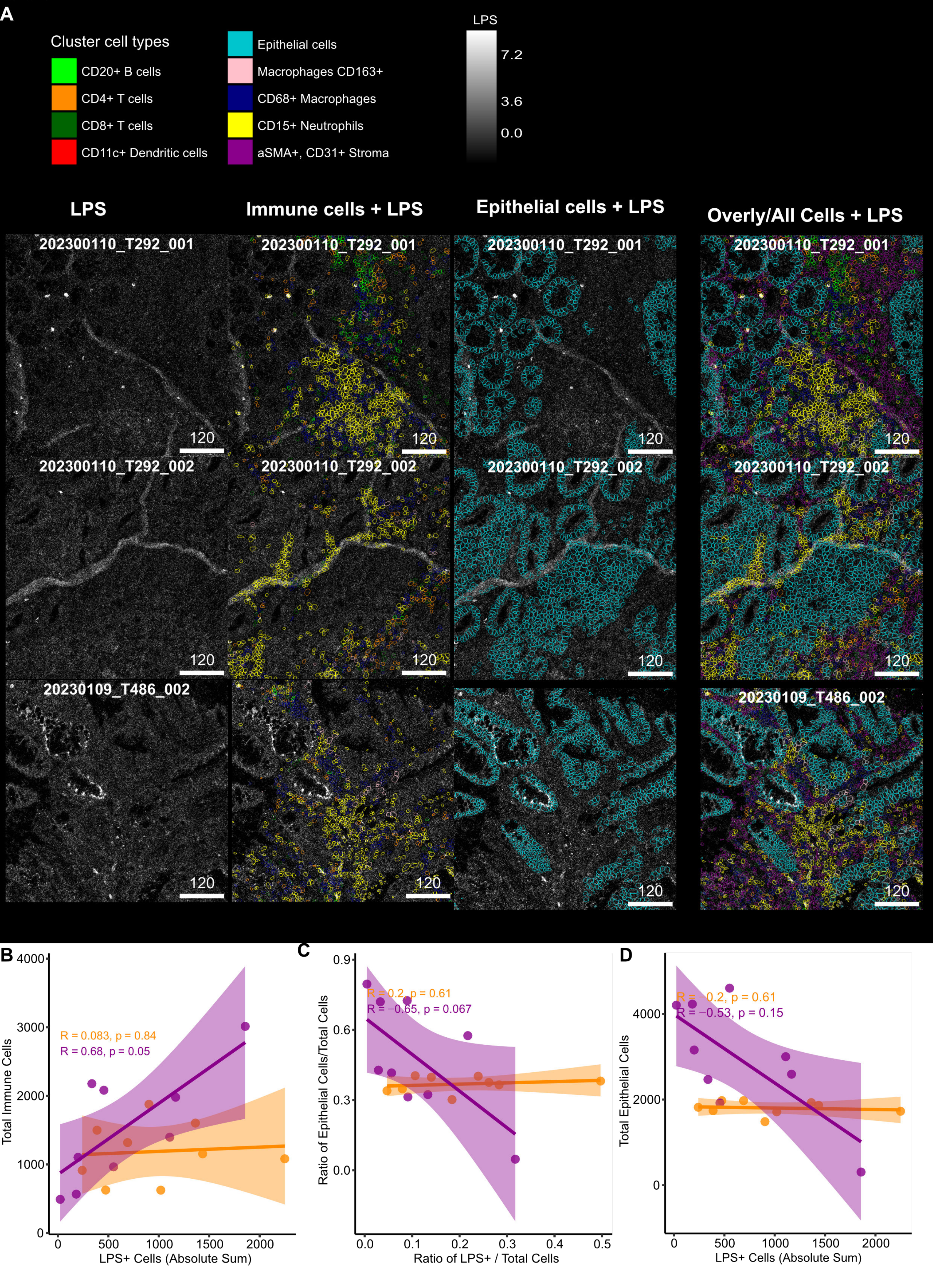

A
Cluster cell types
CD20+ B cells
CD4+ T cells
CD8+ T cells
CD11c+ Dendritic cells
Epithelial cells
Macrophages CD163+
CD68+ Macrophages
CD15+ Neutrophils
aSMA+, CD31+ Stroma
LPS
7.2
3.6
0.0
LPS
Immune cells + LPS
Epithelial cells + LPS
Overly/All Cells + LPS
202300110_T292_001
202300110_T292_002
20230109_T486_002
120
B
R = 0.083, p = 0.84
R = 0.68, p = 0.05
Total Immune Cells
LPS+ Cells (Absolute Sum)
C
R = 0.2, p = 0.61
R = −0.65, p = 0.067
Ratio of Epithelial Cells/Total Cells
Ratio of LPS+ / Total Cells
D
R = −0.2, p = 0.61
R = −0.53, p = 0.15
Total Epithelial Cells
LPS+ Cells (Absolute Sum)

S15

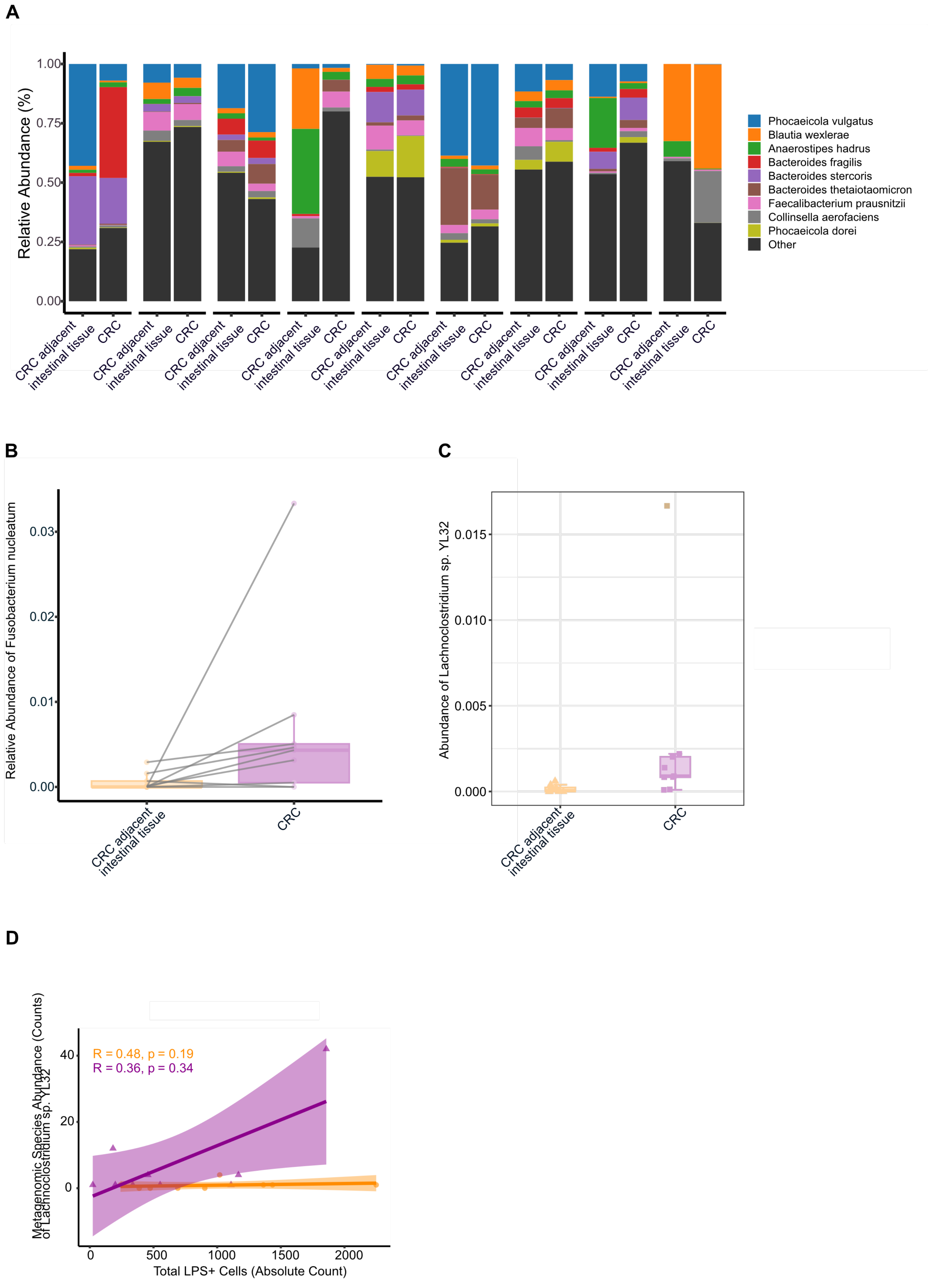
A
Relative Abundance (%)
1.00
0.75
0.50
0.25
0.00
CRC adjacent intestinal tissue
CRC
Phocaeicola vulgatus
Blautia wexlerae
Anaerostipes hadrus
Bacteroides fragilis
Bacteroides stercoris
Bacteroides thetaiotaomicron
Faecalibacterium prausnitzii
Collinsella aerofaciens
Phocaeicola dorei
Other
B
Relative Abundance of Fusobacterium nucleatum
0.03
0.02
0.01
0.00
CRC adjacent intestinal tissue
CRC
C
Abundance of Lachnoclostridium sp. YL32
0.015
0.010
0.005
0.000
CRC adjacent intestinal tissue
CRC
D
Metagenomic Species Abundance of Lachnoclostridium sp. YL32 (Counts)
R = 0.48, p = 0.19
R = 0.36, p = 0.34
40
20
0
0
500
1000
1500
2000
Total LPS+ Cells (Absolute Count)

S16

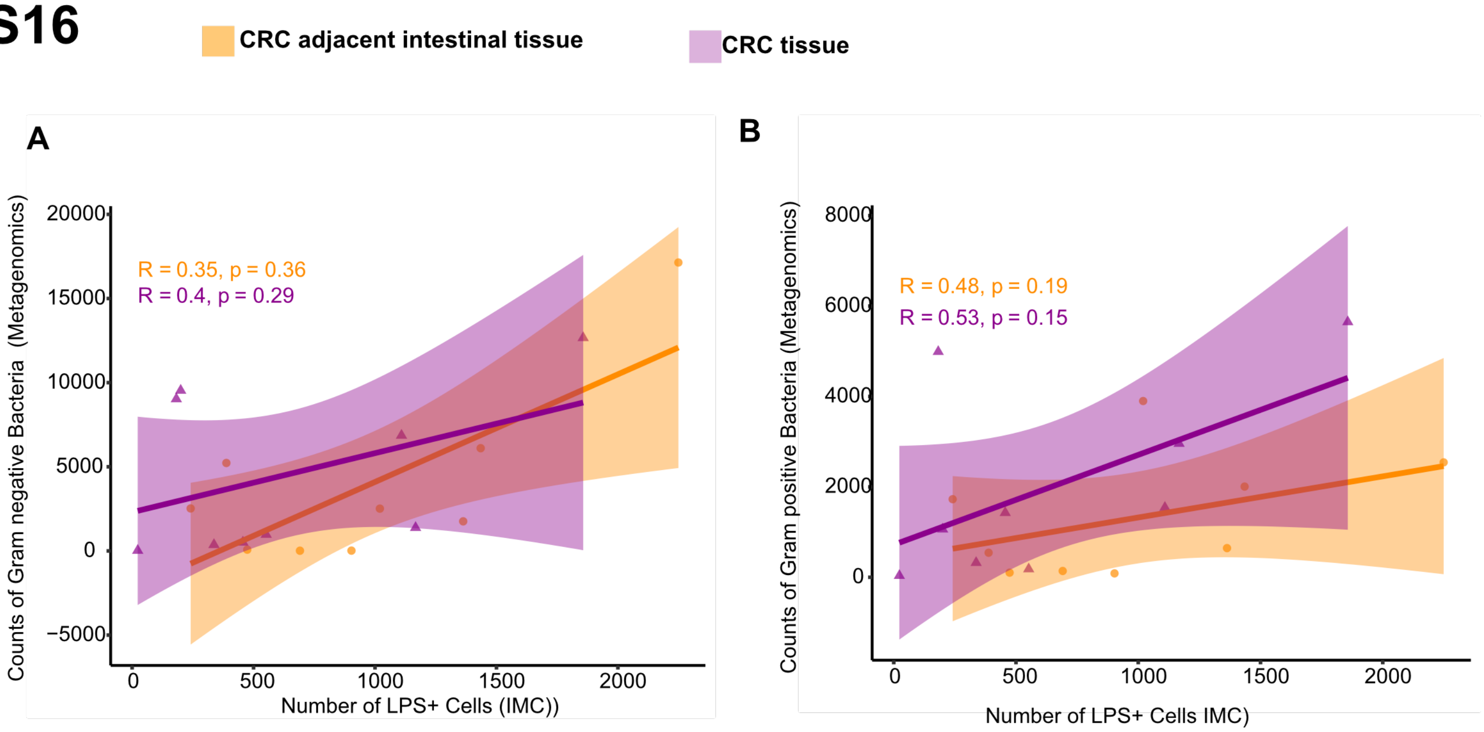
CRC adjacent intestinal tissue
CRC tissue
A
R = 0.35, p = 0.36
R = 0.4, p = 0.29
Counts of Gram negative Bacteria (Metagenomics)
20000
15000
10000
5000
0
−5000
0
500
1000
1500
2000
Number of LPS+ Cells (IMC))
B
R = 0.48, p = 0.19
R = 0.53, p = 0.15
Counts of Gram positive Bacteria (Metagenomics)
8000
6000
4000
2000
0
Number of LPS+ Cells IMC)

S17

A

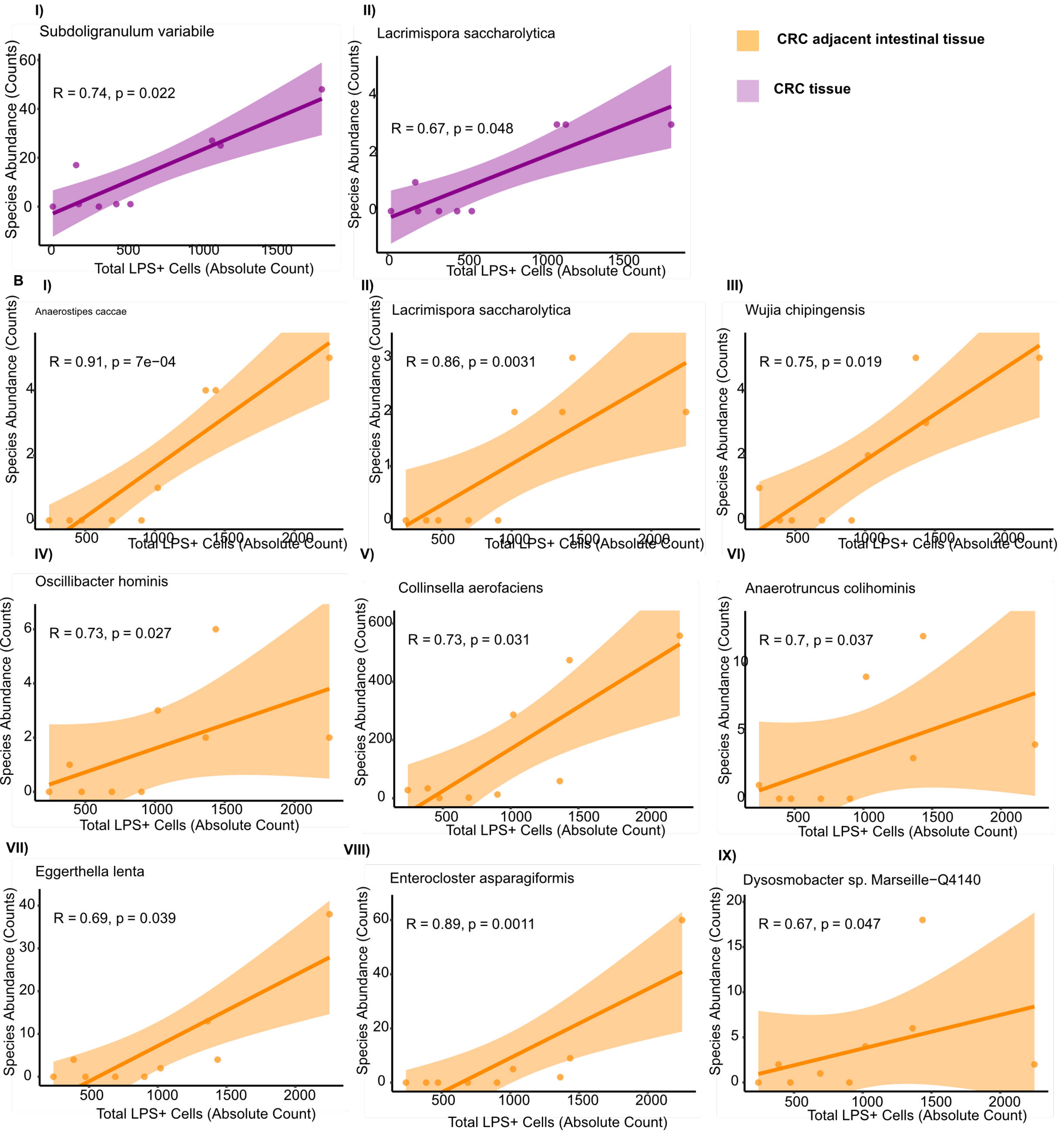
I)
Subdoligranulum variabile
R = 0.74, p = 0.022
Species Abundance (Counts)
Total LPS+ Cells (Absolute Count)
II)
Lacrimispora saccharolytica
R = 0.67, p = 0.048
Species Abundance (Counts)
Total LPS+ Cells (Absolute Count)
CRC adjacent intestinal tissue
CRC tissue
B
I)
Anaerostipes caccae
R = 0.91, p = 7e−04
II)
Lacrimispora saccharolytica
R = 0.86, p = 0.0031
III)
Wujia chipingensis
R = 0.75, p = 0.019
IV)
Oscillibacter hominis
R = 0.73, p = 0.027
V)
Collinsella aerofaciens
R = 0.73, p = 0.031
VI)
Anaerotruncus colihominis
R = 0.7, p = 0.037
VII)
Eggerthella lenta
R = 0.69, p = 0.039
VIII)
Enterocloster asparagiformis
R = 0.89, p = 0.0011
IX)
Dysosmobacter sp. Marseille−Q4140
R = 0.67, p = 0.047
Species Abundance (Counts)
Total LPS+ Cells (Absolute Count)

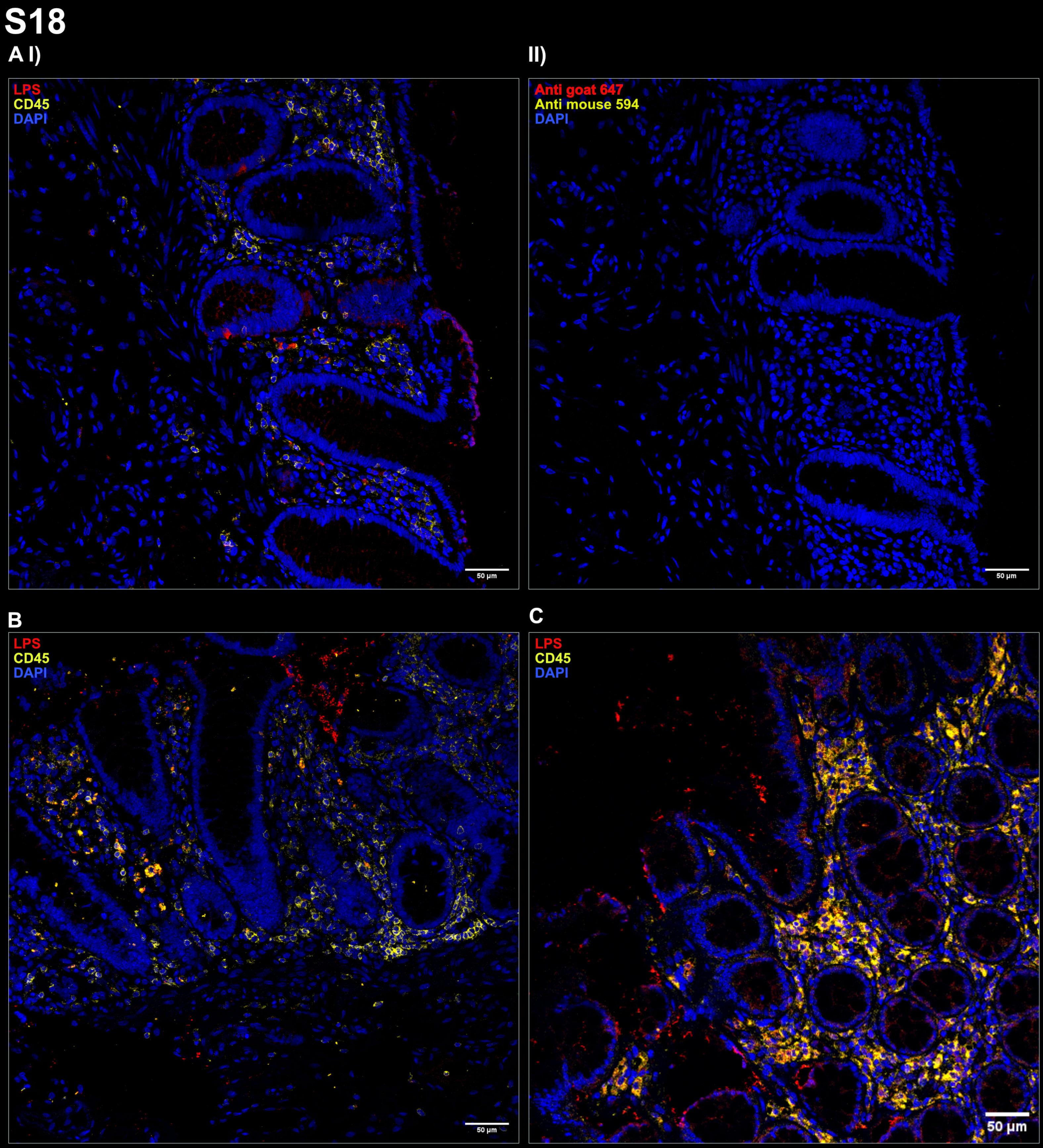
S18
A I)
II)
LPS
CD45
DAPI
50 µm
Anti goat 647
Anti mouse 594
DAPI
50 µm
B
LPS
CD45
DAPI
50 µm
C
LPS
CD45
DAPI
50 µm

S18

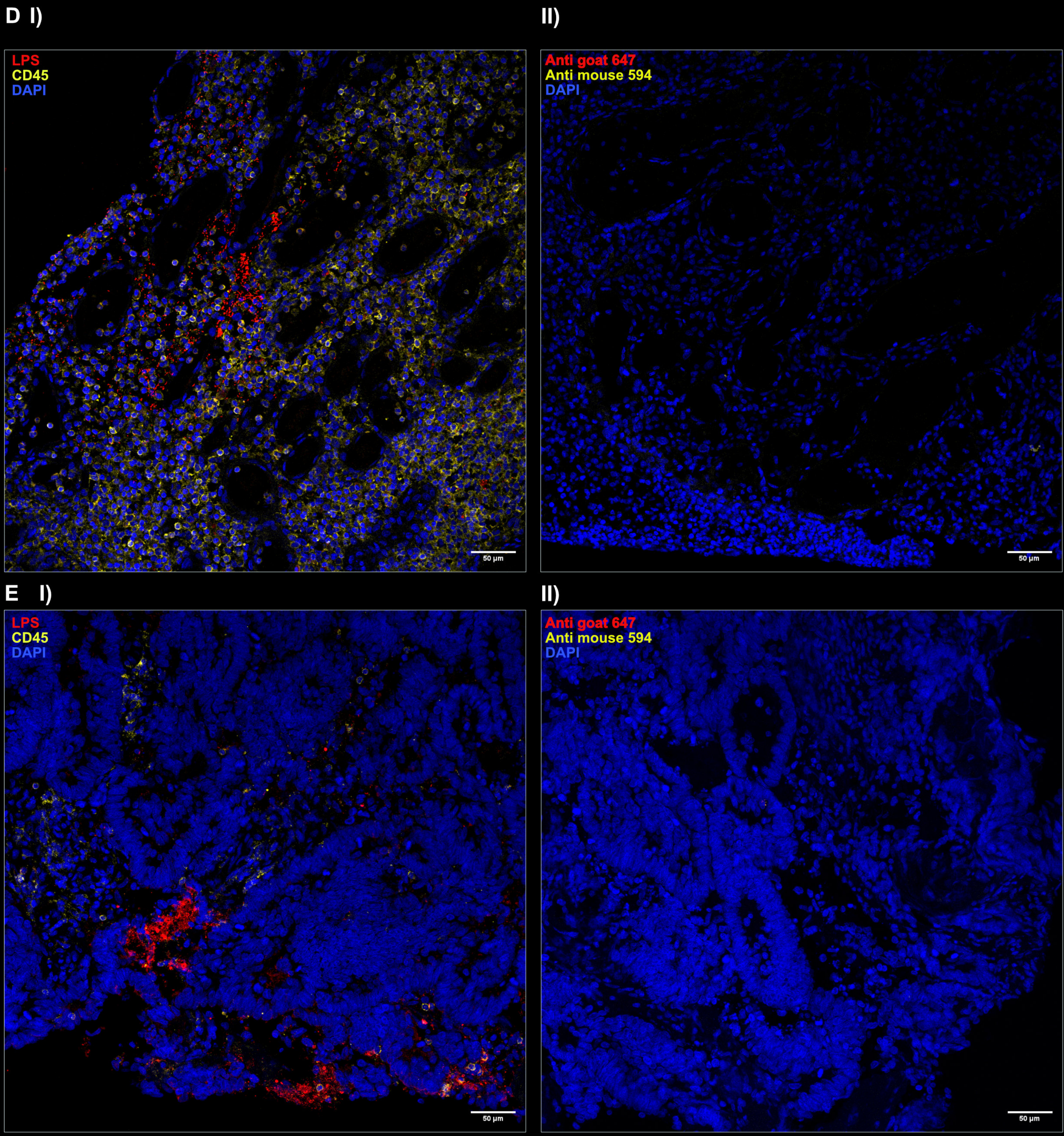
D I)
II)
LPS
CD45
DAPI
Anti goat 647
Anti mouse 594
DAPI
50 µm
50 µm
E I)
II)
LPS
CD45
DAPI
Anti goat 647
Anti mouse 594
DAPI
50 µm
50 µm
